# NAD^+^ exhaustion by CD38 upregulation contributes to blood pressure elevation and vascular damage in hypertension

**DOI:** 10.1038/s41392-023-01577-3

**Published:** 2023-09-18

**Authors:** Yumin Qiu, Shiyue Xu, Xi Chen, Xing Wu, Zhe Zhou, Jianning Zhang, Qiang Tu, Bing Dong, Zhefu Liu, Jiang He, Xiaoyu Zhang, Shuangshuang Liu, Chen Su, Hui Huang, Wenhao Xia, Jun Tao

**Affiliations:** 1 Department of Hypertension and Vascular Disease, The First Affiliated Hospital of Sun Yat-sen University, 510080 Guangzhou, China; 2 National-Guangdong Joint Engineering Laboratory for Diagnosis and Treatment of Vascular Diseases, 510080 Guangzhou, China; 3 Key Laboratory on Assisted Circulation, Ministry of Health, 510080 Guangzhou, China; 4 Department of Cardiology, The Eighth Affiliated Hospital of Sun Yat-sen University, 518033 Shenzhen, China; 5 Guangxi Hospital Division of The First Affiliated Hospital, Sun Yat-sen University, 530022 Nanning, China

**Keywords:** Cardiology, Clinical trials

## Abstract

Hypertension is characterized by endothelial dysfunction and arterial stiffness, which contribute to the pathogenesis of atherosclerotic cardiovascular diseases. Nicotinamide adenine dinucleotide (NAD^+^) is an indispensable cofactor in all living cells that is involved in fundamental biological processes. However, in hypertensive patients, alterations in NAD^+^ levels and their relation with blood pressure (BP) elevation and vascular damage have not yet been studied. Here we reported that hypertensive patients exhibited lower NAD^+^ levels, as detected by high-performance liquid chromatography-mass spectrometry (HPLC-MS), in both peripheral blood mononuclear cells (PBMCs) and aortas, which was parallel to vascular dysfunction. NAD^+^ boosting therapy with nicotinamide mononucleotide (NMN) supplement reduced BP and ameliorated vascular dysfunction in hypertensive patients (NCT04903210) and AngII-induced hypertensive mice. Upregulation of CD38 in endothelial cells led to endothelial NAD^+^ exhaustion by reducing NMN bioavailability. Pro-inflammatory macrophages infiltration and increase in IL-1β generation derived from pro-inflammatory macrophages resulted in higher CD38 expression by activating JAK1-STAT1 signaling pathway. CD38 KO, CD38 inhibitors treatment, or adeno-associated virus (AAV)-mediated endothelial CD38 knockdown lowered BP and improved vascular dysfunction in AngII-induced hypertensive mice. The present study demonstrated for the first time that endothelial CD38 activation and subsequently accelerated NAD^+^ degradation due to enhanced macrophage-derived IL-1β production was responsible for BP elevation and vascular damage in hypertension. NAD^+^ boosting therapy can be used as a novel therapeutic strategy for the management of hypertensive patients.

## Introduction

Hypertension represents a major public health issue that is responsible for 8.5 million deaths due to stroke, ischemic heart disease and renal disease worldwide.^1^ Endothelial dysfunction and arterial stiffness are considered hallmarks of hypertension and play an essential role in the pathogenesis of atherosclerotic cardiovascular diseases.^2–4^ Studies have demonstrated that improvements in vascular function alleviate the development of morphological atherosclerotic changes and contribute to the reduction of later clinical complications.^5–8^ Therefore, further understanding of the specific molecular mechanisms of hypertension and its associated vascular damage is of great clinical significance in seeking an innovative therapeutic strategy for the management of hypertension.

Nicotinamide adenine dinucleotide (NAD^+^) is a central cofactor involved in cellular biological activities that declines during aging.^9–12^ Restoring NAD^+^ levels with dietary supplement and regulating NAD^+^ metabolic enzymes with small molecular compounds have emerged as a potential therapeutic measure to elevate NAD^+^ concentrations, thereby providing opportunities for ameliorating aging.^13–16^ In aged mice, NAD^+^ administration improved endothelial-mediated vasorelaxation and reduced arterial stiffness.^17^ In the healthy elderly, a recent clinical study revealed that supplement of nicotinamide riboside (NR), an NAD^+^ precursor, mitigated arterial stiffness as well.^18^ Given its pivotal role in cellular processes, the association between NAD^+^ levels and human health has been definitely established. Hypertension is acknowledged as a kind of aging-related disease and NAD^+^ supplement may be a promising clinical strategy as an aging-targeted intervention for hypertension. However, changes in NAD^+^ levels and their relationship with blood pressure (BP) elevation and vascular damage have not yet been explored in patients with hypertension.

NAD^+^ levels are determined by the dynamic balance of biosynthesis and degradation.^19^ CD38, which was first reported as a multifunctional protein with both glycohydrolase and ADP-ribosyl cyclase activities, has recently been recognized as a key rate-limiting enzyme of NAD^+^ and nicotinamide mononucleotide (NMN) degradation.^20–22^ Inhibition of CD38 not only prevented age-related declines in NAD^+^ levels, ameliorated metabolic dysfunction, and reduced DNA damage accumulation,^23^ but also protected against postischemic endothelial injury in mice.^24^ However, in patients with hypertension, the association of CD38 expression with NAD^+^ levels is unclear. It is generally accepted that hypertension is a kind of chronic inflammatory disease.^25^ Macrophage infiltration into the vascular endothelial layer and the subsequent release of a series of cytokines, such as IL-1β, contributes to this pro-inflammatory status. Interestingly, recent evidence has demonstrated that inflammation is involved in the regulation of CD38 expression.^26^ However, whether the change in endothelial CD38 is mediated by macrophage-derived IL-1β in hypertension is still unknown.

In this study, we hypothesized that NAD^+^ exhaustion in endothelial cells mediated by CD38 activation contributed to the BP elevation and vascular damage in hypertension. Moreover, the inflammation induced by macrophage infiltration plays a key role in the CD38 activation, promoting NAD^+^ deprivation in hypertension. To address these issues, NAD^+^ levels were detected in the peripheral blood mononuclear cells (PBMCs) and aortas of hypertensive patients, and their association with BP and vascular dysfunction was analyzed. Moreover, a randomized control study was carried out to investigate the effect of NMN supplement on BP and vascular function in patients with hypertension. Mechanistically, we explored the effect of macrophage-endothelium interactions on CD38 and NAD^+^ levels in hypertension in vitro and in vivo.

## Results

### The decrease in NAD^+^ levels in PBMCs associated with vascular dysfunction is restored by NMN supplement in patients with hypertension

**Fig. 1:**
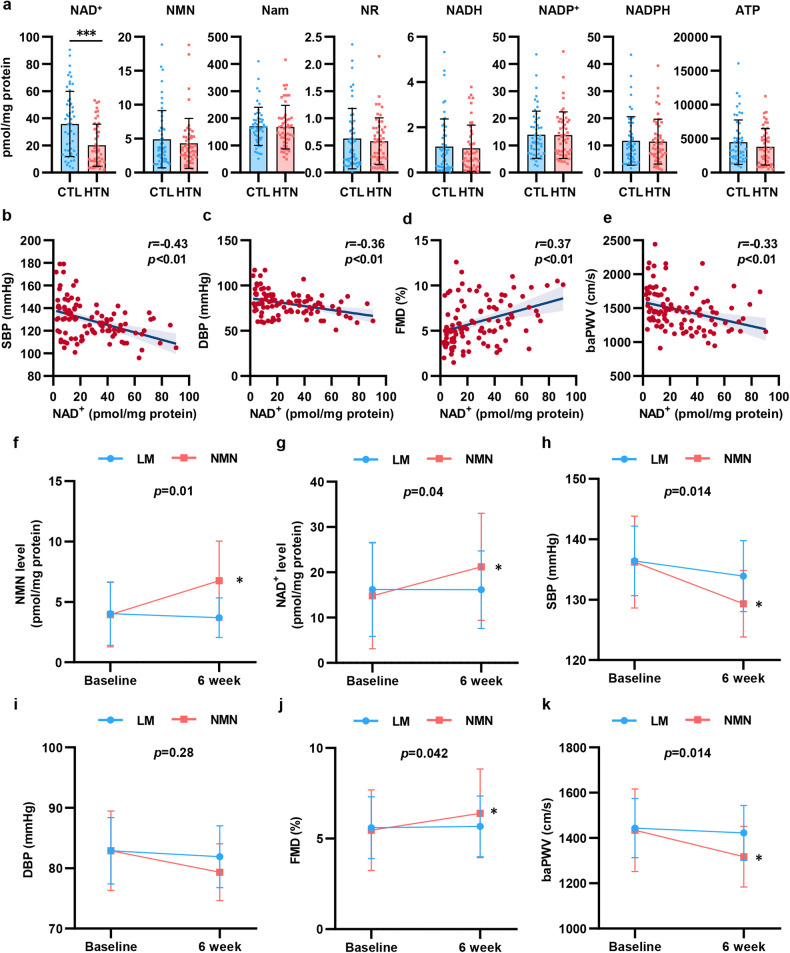
Decline in NAD^**+**^ level from PBMCs associated with vascular function was restored by NMN supplement in hypertension. **a** NAD^+^ and its metabolites levels in PBMCs were detected by HPLC-MS in hypertensive patients (*n* = 50) and healthy subjects (*n* = 52). **b**, **c** NAD^+^ level in PBMCs were correlated with SBP (**b**) and DBP (**c**). **d**, **e** NAD^+^ level in PBMCs were correlated with FMD (**d**) and baPWV (**e**). **f**–**j** Changes NMN level (**f**), NAD^+^ level (**g**), systolic blood pressure (**h**), diastolic blood pressure (**i**), FMD (**j**), and baPWV (**k**) from baseline to the end of treatment (Life modification [LM] group: *n* = 10; NMN supplement group: *n* = 9). *p < 0.05, ***p < 0.001

To explore the change in NAD^+^ levels in hypertension, we enrolled 102 participants, including 52 healthy subjects and 50 patients who were newly diagnosed with hypertension. We isolated PBMCs from the participants and detected NAD^+^ levels and metabolite levels by high-performance liquid chromatography-mass spectrometry (HPLC-MS). As shown in Fig. 1a, NAD^+^ levels were significantly decreased by 44% in PBMCs isolated from hypertensive patients. To assess the association between NAD^+^ levels and BP elevation and vascular function in hypertension, we evaluated vascular function by measuring flow mediated dilation (FMD) of the branchial artery and branchial-ankle pulse wave velocity (baPWV). As shown in Supplementary Fig. S1, hypertensive patients displayed decreased FMD and increased baPWV, which indicated vascular damage in hypertension. In addition, we found that there was a negative correlation between BP and NAD^+^ levels in PBMCs (Fig. 1b, c). Of note, NAD^+^ levels were found to be related to FMD and baPWV (Fig. 1d, e). In the subgroup analysis stratified by BP, there was a more obvious association between the NAD^+^ level and FMD and baPWV in hypertensive patients than in healthy subjects (Supplementary Fig. S2). These results suggested that decreased NAD^+^ levels may be involved in the modulation of hypertension and vascular dysfunction.

To further demonstrate the essential role of NAD^+^ in hypertension, patients initially diagnosed with mild essential hypertension (BP ranged from 130/80 to 159/99 mmHg)^27^ ranging from 18 to 80 years in age were enrolled in the study. Among the 24 screened participants, 21 met the inclusion criteria, and were randomly assigned to treatment with life modification plus NMN, an NAD^+^ precursor (NMN group) or life modification only (LM group). Two participants withdrew prematurely from the trial, which resulted in 19 participants finishing the study (*n* = 9, NMN group; *n* = 10, LM group; Supplementary Fig. S3).

The baseline characteristics of the 2 groups of hypertensive patients were comparable, as shown in Table 1. During the trial, NMN was well tolerated, and no adverse events occurred (Supplementary Table S1). We assessed the NAD^+^ metabolites in PBMCs before and after intervention. Oral NMN supplement effectively increased NAD^+^ levels in PBMCs by ~43% compared with the LM group (Fig. 1f, g and Supplementary Table S2). Additionally, we also observed an increase in energy production and metabolism, including adenosine and adenosine triphosphate (ATP), however, there was no statistical significance mainly due to the small sample size. Collectively, these findings showed that NMN supplement effectively improved NAD^+^ metabolism in hypertensive patients.

**Table 1:** Effects of NMN on BP, metabolic parameters and vascular function

	LM (*n* = 10)			NMN (*n* = 9)			*P*_difference_
	Baseline	6 weeks	*P*	Baseline	6 weeks	*P*	
Demographics							
Age (years)	46.70 ± 11.19	46.70 ± 11.19	–	46.00 ± 12.86	46.00 ± 12.86	–	0.40
Male sex [*n* (%)]	5 (50.00%)	5 (50.00%)	–	4 (44.44%)	4 (44.44%)	–	0.81
Body weight (kg)	62.90 ± 10.08	62.80 ± 9.84	0.59	62.72 ± 22.91	62.67 ± 7.78	0.84	0.89
BMI (kg/m^2^)	22.92 ± 1.86	22.89 ± 1.77	0.61	22.91 ± 3.41	22.88 ± 3.46	0.80	0.96
Smoking [*n* (%)]	1 (10.00%)	1 (10.00%)	–	1 (11.11%)	1 (11.11%)	–	0.94
BP							
SBP (mmHg)	136.40 ± 5.78	133.90 ± 5.90	0.07	136.22 ± 7.61	129.33 ± 5.50	<0.001	0.014
DBP (mmHg)	82.90 ± 5.51	81.91 ± 5.11	0.51	82.89 ± 6.57	79.33 ± 4.72	0.08	0.28
Metabolic indices							
Fasting serum glucose (mmol/L)	5.02 ± 0.57	5.09 ± 0.81	0.83	4.48 ± 0.35	4.34 ± 0.28	0.46	0.59
Total cholesterol (mmol/L)	4.57 ± 0.76	4.63 ± 0.76	0.75	5.39 ± 1.28	5.54 ± 1.09	0.66	0.80
Triglyceride (mmol/L)	1.67 ± 1.37	1.55 ± 1.40	0.65	1.22 ± 0.68	1.08 ± 0.47	0.41	0.93
HDL cholesterol (mmol/L)	1.36 ± 0.36	1.40 ± 0.35	0.37	1.34 ± 0.24	1.34 ± 0.27	0.94	0.63
LDL cholesterol (mmol/L)	2.69 ± 0.55	2.78 ± 0.51	0.48	3.49 ± 0.94	3.62 ± 0.82	0.62	0.89
ALT (U/L)	23.50 ± 14.45	22.90 ± 8.75	0.86	19.89 ± 12.73	23.33 ± 14.65	0.42	0.45
AST (U/L)	23.80 ± 8.94	26.20 ± 11.16	0.60	21.44 ± 6.88	24.78 ± 7.87	0.18	0.86
Creatinine (μmol/L)	71.20 ± 25.62	71.40 ± 22.81	0.93	73.89 ± 16.52	74.67 ± 16.94	0.75	0.86
Uric acid (μmol/L)	358.60 ± 98.45	328.80 ± 116.39	0.13	339.44 ± 79.12	336.33 ± 55.09	0.85	0.28
Vascular function							
FMD (%)	5.60 ± 1.71	5.67 ± 1.67	0.72	5.46 ± 2.23	6.39 ± 2.45	0.031	0.042
baPWV (cm/s)	1443.50 ± 130.55	1422.70 ± 121.38	0.19	1434.22 ± 182.23	1317.56 ± 134.41	0.006	0.01
Inflammatory indices							
Hs-CRP (mg/L)	0.92 ± 0.74	0.84 ± 0.71	0.09	1.16 ± 1.18	0.88 ± 0.73	0.54	0.64
Serum IL-1β (ng/L)	0.34 ± 0.24	0.34 ± 0.26	0.92	0.29 ± 0.19	0.22 ± 0.14	0.049	0.28
Serum IL-6 (ng/L)	14.03 ± 13.24	13.04 ± 11.24	0.53	13.26 ± 17.82	11.09 ± 15.87	0.14	0.57
Serum IL-18 (ng/L)	12.43 ± 11.39	12.10 ± 9.41	0.75	12.50 ± 12.40	11.75 ± 8.50	0.77	0.87

Two-sided paired *t* test or Wilcoxon signed rank test was used for the comparison within groups. Independent sample t test was performed in the comparisons of difference of two group. Continuous variables are expressed as mean ± SD for parametric variables and as median (interquartile range) for non-parametric variables. *P*_difference_ in age, male sex, smoking indicates the *P* value of difference of baseline while the other *P*_difference_ indicates the *P* value of differences before and after intervention between LM and NMN group

*LM* life modification, *SBP* systolic blood pressure, *DBP* diastolic blood pressure, *HbA*_*1C*_ glycated hemoglobin, *HDL* high-density lipoprotein, *LDL* low-density lipoprotein, *IL* interleukin, *TNF* tumor necrosis factor, *FMD* flow mediated dilation, *baPWV* brachial ankle pulse wave velocity

More importantly, our results showed a beneficial effect of NMN on BP and vascular function. NMN supplement for 6 weeks reduced SBP and DBP by 6.11 mmHg and 3.56 mmHg, respectively, with a significant difference compared with the LM group (Fig. 1h, i and Table 1). In addition to the reduction in BP, FMD increased by 0.6%, while baPWV decreased by 116.66 cm/s after 6 weeks of NMN supplement (Fig. 1j, k and Table 1). Our data proved that NMN supplement reduced BP and ameliorated vascular damage in hypertensive patients.

In addition, we performed in vitro experiments to determine the protective function of NMN on endothelial function. AngII stimulation reduced the level of p-eNOS (endothelial NO synthase), which was rescued by NMN supplement, as well as the level of NO in the medium (Supplementary Fig. S4).

### NAD^+^ levels in aortas from hypertensive patients and angll-induced hypertensive mice are reduced

**Fig. 2:**
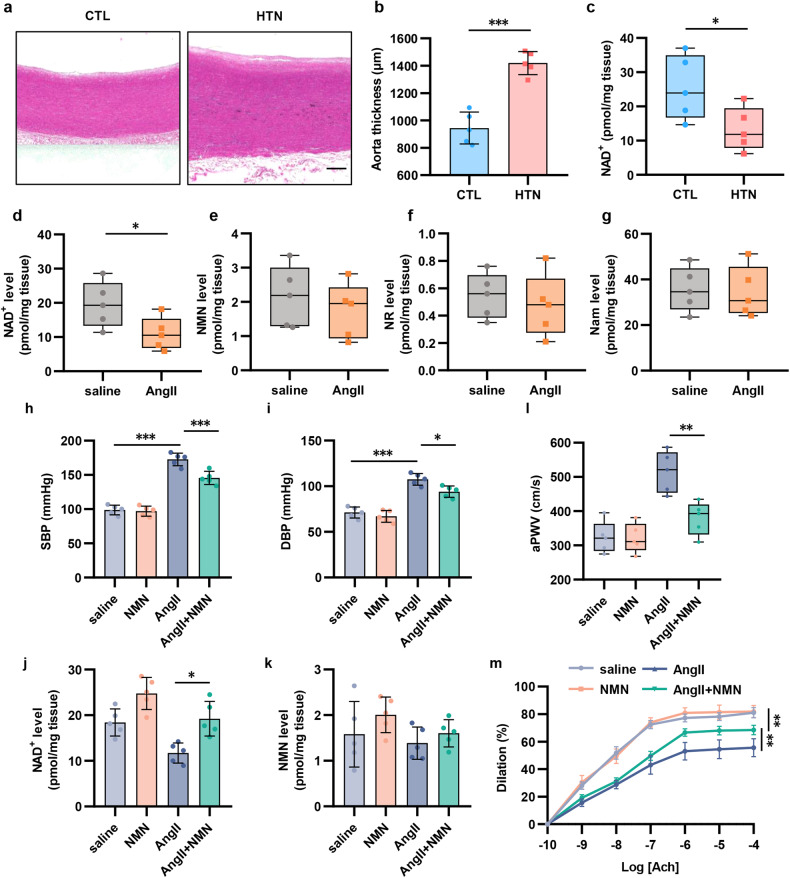
NAD^**+**^ level of aortas from hypertensive patients and AngII-induced hypertensive mice is reduced. **a**, **b** Representative images of thoracic aorta sections from hypertensive patients (*n* = 5) and healthy subjects (*n* = 5) stained with Von Kossa. Scale bar = 250 μm. **c** NAD^+^ level was measured in thoracic aorta of hypertensive patients and healthy subjects (*n* = 5). **d**–**g** NAD^+^, NMN, NR and Nam levels in mice aortas were detected by HPLC-MS (*n* = 5). **h**, **i** The change of SBP and DBP was detected by a tail-cuff plethysmography system (*n* = 5). **j**, **k** NAD^+^ and NMN levels in mice aortas of different groups were detected by HPLC-MS (*n* = 5). **l** In vivo aortic pulse wave velocity was measured by Doppler (*n* = 5). **m** The aortic ring assay was performed to evaluate the Ach-vasodilation (*n* = 5). *p < 0.05, **p < 0.01, ***p < 0.001

Although NAD^+^ levels declined in PBMCs in hypertensive patients, whether the change in NAD^+^ in the arterial wall directly participated in the regulation of BP and vascular function should be further investigated. Therefore, NAD^+^ levels in the aortic tissues of hypertensive patients and healthy controls were detected. First, we used Von Kossa staining and confirmed the increased thickness and obvious calcification in hypertensive aortas (Fig. 2a, b). Then, we found that the NAD^+^ levels of hypertensive aortas were significantly reduced by 47.7% (Fig. 2c and Supplementary Fig. S5).

To further confirm whether the decline in human aortic NAD^+^ was related to BP elevation and vascular damage, a hypertensive mice model was established by 4 weeks of subcutaneous administration of AngII through osmotic pumps (Supplementary Fig. S6a–d). Similar to hypertensive patients’ aortas, the aortas of hypertensive mice exhibited a 43.7% reduction in NAD^+^ levels (Fig. 2d–g), which was accompanied by worse aortic PWV (aPWV) and Ach-induced vasorelaxation (Supplementary Fig. S6e, f).

To further reveal the key role of arterial NAD^+^ in BP and vascular function, hypertensive mice were treated with NMN supplement by oral administration for 28 days (Supplementary Fig. S7a). Consistent with the clinical study, both SBP and DBP declined after NMN supplement in hypertensive mice (Fig. 2h, i). Furthermore, HPLC-MS analyses showed increased levels of aortic NAD^+^ in mice supplemented with NMN (Fig. 2j, k). Next, endothelial-dependent vasodilation (Ach-induced and SNP-induced vasorelaxation) and aPWV were measured to assess vascular function. NMN supplement significantly improved endothelial function and arterial stiffness in hypertensive mice (Fig. 2l, m and Supplementary Fig. S7e). Furthermore, hematoxylin-eosin (HE), Masson and Verhoeff’s Van Gieson (EVG) staining were used to explore the effect of NMN supplement on adverse vascular remodeling. NMN supplement attenuated AngII-induced vascular remodeling including increased media thickness and media-to-lumen ratio (Supplementary Fig. S7b–d). Taken together, these findings further confirmed that arterial NAD^+^ levels were essential for controlling BP and vascular function in hypertension.

### Endothelial CD38 plays a key role in the decreased of NAD^+^ level by controlling NMN bioavailability

**Fig. 3:**
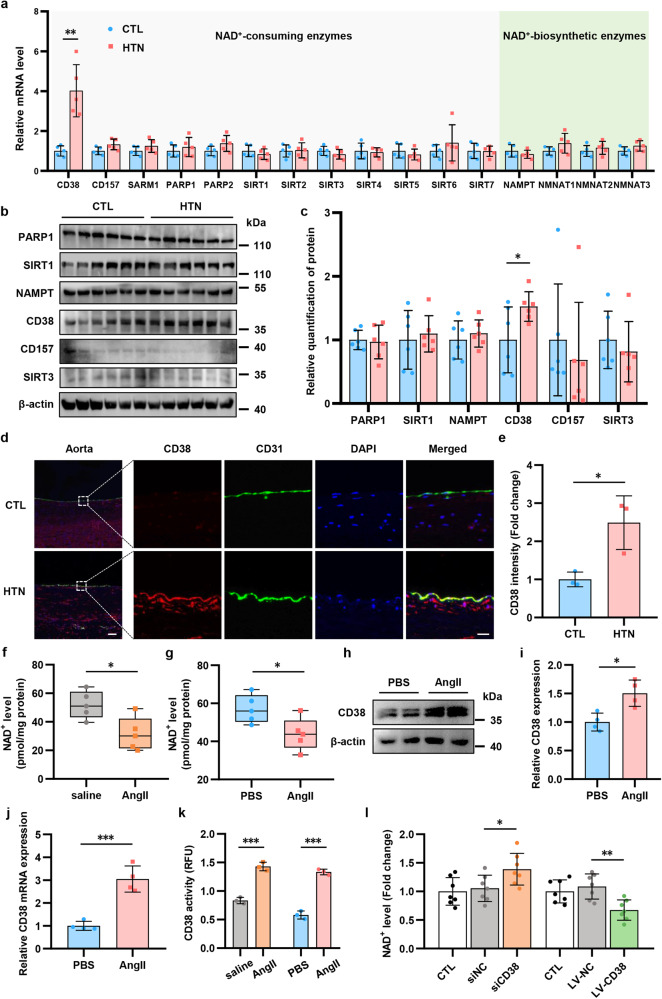
CD38 is required for endothelial cells NAD^**+**^ decline in hypertension. **a** mRNA levels of NAD-consuming and NAD-biosynthetic enzymes were measured using quantitative PCR in human aortas (*n* = 5). **b**, **c** Western analysis of NADase enzymes in human aortas (*n* = 6). **d**, **e** Immunofluorescence of the CD38 (red), endothelial cells marker CD31 (green) and DAPI-stained nuclei (blue) in aorta from hypertensive patients and healthy subjects (*n* = 3). Scale bar (original) = 250 μm. Scale bar (magnified) = 25 μm. **f** NAD^+^ level of MAECs was detected by HPLC-MS (*n* = 5). **g** NAD^+^ level was tested in HAECs with or without AngII treatment (100 μM) (*n* = 5). **h**, **i** Western analysis of CD38 in HAECs treated with or without AngII (*n* = 6). **j** mRNA level of CD38 was measured by quantitative PCR in HAECs (*n* = 6). **k** NADase activity was measured in MAECs of hypertensive mice and AngII-induced hypertensive HAECs (*n* = 3). **l** Endothelial cells were transfected with CD38 and negative control siRNA, or vector lentiviruses and CD38 lentiviruses, respectively. NAD^+^ level was detected by HPLC-MS (*n* = 6). *p < 0.05, **p < 0.01, ***p < 0.001

NAD^+^ levels are determined by the balance of biosynthesis and degradation. Therefore, to better understand the mechanisms of NAD^+^ decline, we measured the levels of the enzymes that were involved in the biosynthesis or metabolism of NAD^+^. The mRNA and protein levels of CD38, a rate-limiting NAD^+^ degradation enzyme, were significantly upregulated in human hypertensive aortas (Fig. 3a–c). Considering that endothelial cells play an important role in the regulation of BP and vascular function,^28,29^ we focused on the expression of CD38 in endothelial cells. Immunofluorescence (IF) staining was used to visualize the upregulation of CD38 in endothelial cells in hypertensive aortas compared to the healthy controls (Fig. 3d, e). Similarly, in hypertensive mice, endothelial CD38 was also increased (Supplementary Fig. S8). Next, we isolated mouse primary aortic endothelial cells (MAECs) and found that the NAD^+^ levels in hypertensive MAECs were reduced compared to that in healthy MAECs (Fig. 3f). Then, we further demonstrated a decrease in NAD^+^ levels in AngII-stimulated human aortic endothelial cells (HAECs) in vitro (Fig. 3g). Moreover, qPCR and western blotting were used to show that CD38 increased at both the mRNA and protein levels in AngII-treated HAECs (Fig. 3h–j). In addition to CD38 expression, hypertensive MAECs, and AngII-induced HAECs exhibited higher CD38 NADase activity than the control group (Fig. 3k).

We then investigated the role of CD38 in NAD^+^ homeostasis in HAECs through in vitro gain- and loss-of-function experiments by using specific CD38 siRNA knockdown or a lentivirus encoding human CD38 overexpression system (Supplementary Fig. S9). CD38 knockdown led to the upregulation of NAD^+^ levels whereas CD38 overexpression decreased NAD^+^ levels in HAECs (Fig. 3l).

**Fig. 4:**
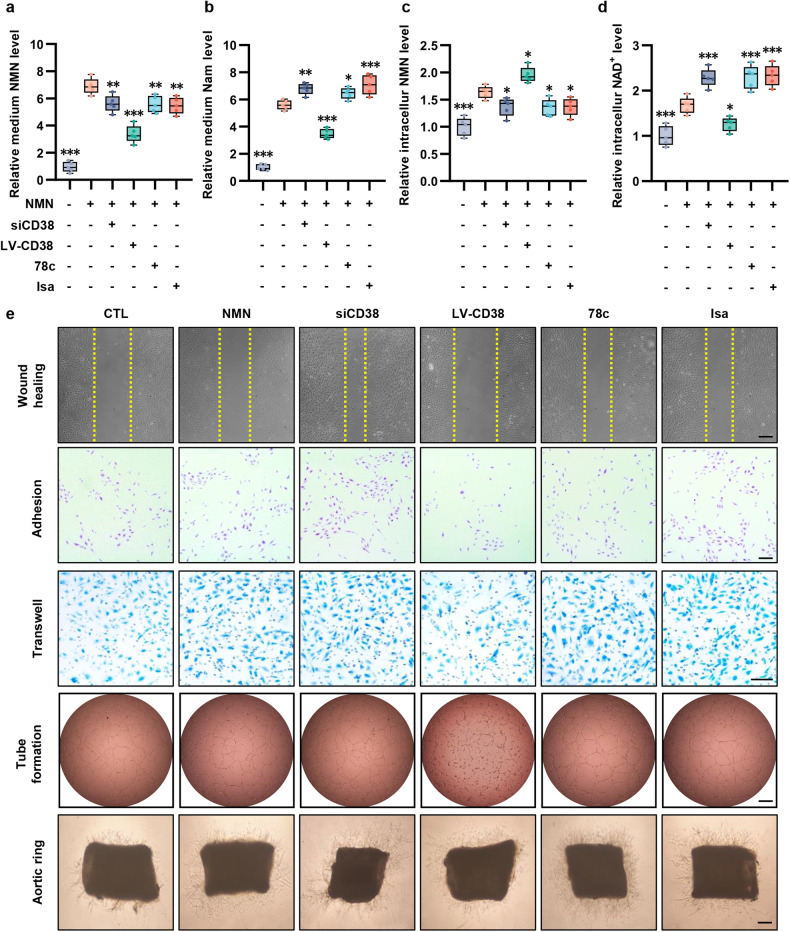
**The ecto-enzymatic activity of CD38 regulates availability of extracellular NMN to endothelial cells. a-d** NAD^+^, Nam and NMN levels inside or outside the endothelial cells co-cultured with NMN, CD38 siRNA, CD38 lentiviruses, 78c and isatuximab (*n* = 5). The differences were compared to NMN group. Scale bar = 100 μm. **e** Endothelial function including would healing, adhesion, transwell, tube formation and aortic ring assays in endothelial cells with different treatment. *p < 0.05, **p < 0.01, ***p < 0.001

In addition to its NADase ability inside cells, CD38 is also reported to catalyze NMN outside of cells through ecto-enzymic activity with a type II membrane orientation.^30^ To determine the contribution of CD38 to NAD^+^ exhaustion, HAECs were incubated with NMN and treated with siCD38, LV-CD38, the CD38 inhibitor 78c, which targets all activity, or the CD38 inhibitor isatuximab, which targets ectoenzymatic activity. HAECs transfected with LV-CD38 showed markedly reduced NMN levels in the culture medium compared to cells treated with siCD38, 78c, and isatuximab (Fig. 4a). Moreover, the level of the NMN degradation product nicotinamide (Nam) in the culture medium of LV-CD38 group was elevated (Fig. 4b). Next, the intracellular levels of NAD^+^ and NMN were measured. Our data confirmed that intracellular NAD^+^ and NMN levels were increased when CD38 was blocked, and this effect was reversed by LV-CD38 transfection (Fig. 4c, d). Notably, treating cells with isatuximab appeared to have the same effect as 78c (Fig. 4a–d). Next, we investigated the association between NAD^+^ levels and endothelial function in response to different CD38 treatment. As depicted in Fig. 4e and Supplementary Fig. S10a–c, the wound-healing, adhesion, and transwell assays showed that the inhibition of CD38 with siCD38, 78c, and isatuximab significantly enhanced endothelial function compared with the NMN group or LV-CD38 group, which was consistent with previous reports that the NAD^+^ level plays an important role in EC homeostasis. Moreover, the in vitro tube formation and aortic ring assays also demonstrated that the inhibition of CD38 showed a favorable effect on endothelial angiogenetic capacity (Supplementary Fig. S10d, e). All these results demonstrated that the ecto-enzymatic activity of CD38 was essential in the regulation of intracellular NAD^+^ levels and endothelial function.

### Infiltrated macrophage-derived IL-1β promotes CD38 transcription via JAK1/STAT1 signaling pathway

**Fig. 5:**
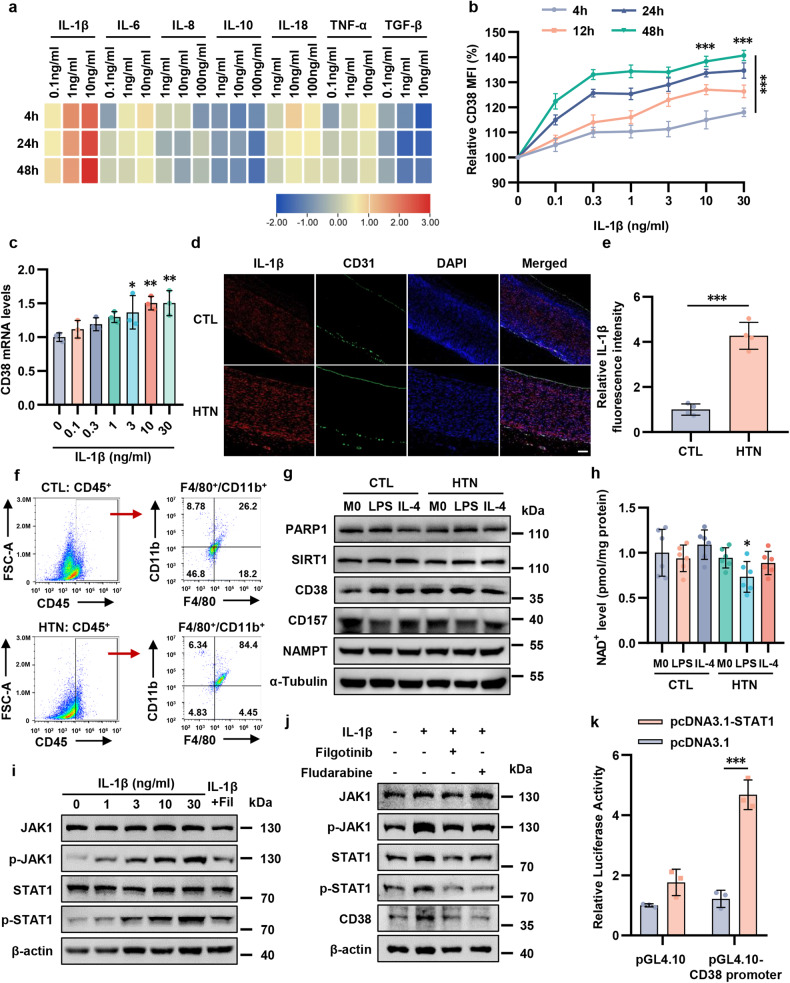
IL-1β derived from pro-inflammatory macrophages promoted CD38 transcription via JAK1/STAT1 pathway. **a** Endothelial cells were cultured with control culture medium or culture medium containing the indicated cytokines (IL-1β, IL-6, IL-8, IL-10, IL-18, TNF-α and TGF-β). CD38 expression was detected by flow cytometry after 4-hour, 24-hour and 48-hour incubation, and relative CD38 MFI was calculated in comparison with MFI of control. Heatmap was constructed based on relative CD38 MFI. **b** Endothelial cells were treated for 48 h with IL-1β (0.1–30 ng/mL), and CD38 expression was measured by flow cytometry. Two-way ANOVA was used for the analysis of differences. The differences in the figures represented significant differences in time and concentration effect. The differences were compared to the control group. **c** mRNA level of CD38 was measured in endothelial cells treated for 48 h with IL-1β (0.1–30 ng/mL) (*n* = 3). The differences were compared to IL-1β 0 ng/mL group. **d**, **e** Immunofluorescence of the IL-1β (red), endothelial cells marker CD31 (green) and DAPI-stained nuclei (blue) in aorta from hypertensive patients and healthy subjects (*n* = 4). Scale bar = 250 μm. **f** The frequency of CD45^+^F4/80^+^CD11b^+^ cells subsets in aortas isolated from hypertensive mice or control mice and analyzed by flow cytometry. **g** Representative immunoblotting showing CD38 expression in endothelial cells co-cultured with cell culture supernatant of M0, pro-inflammatory macrophages and anti-inflammatory macrophages (*n* = 3). **h** NAD^+^ level of endothelial cells was detected by HPLC-MS (*n* = 6). The differences were compared to CTL-M0 group. **i** Representative immunoblotting of JAK1, p-JAK1, STAT1 and p-STAT1 protein levels. **j** The expression of JAK1, p-JAK1, STAT1, and p-STAT1 protein levels in endothelial cells treated with IL-1β, JAK1 inhibitor Filgotinib, and STAT1 inhibitor Fludarabine were detected by western blot assay. **k** Endothelial cells transfected with plasmids were measured by luciferase assay to survey the interaction between STAT1 and CD38 (*n* = 3). *p < 0.05, **p < 0.01, ***p < 0.001

Hypertension is an inflammatory disease characterized by macrophage infiltration into the vascular wall and the release of numerous inflammatory cytokines.^25^ Therefore, the mRNA level and protein expression of inflammatory cytokines were measured in human aortas and the results showed that hypertensive aortas exhibited an inflammatory condition (Supplementary Fig. S11). Recently, CD38 has been reported to be affected by inflammatory cytokines.^26^ Thus, to identify the potential inflammatory cytokines related to CD38 expression in ECs in vitro, we measured the effect of a series of cytokines (IL-1β, IL-6, IL-8, IL-10, IL-18, TNF-α, and TGF-β) on CD38 by flow cytometry. Notably, IL-1β triggered the most significant increase in CD38 expression (Fig. 5a). Our study further showed that the increase in CD38 on ECs was triggered by IL-1β in a dose-dependent and a time-dependent manner (Fig. 5b, c). Moreover, IF assay further demonstrated that IL-1β was accumulated in hypertensive aortas in both humans and mice (Fig. 5d, e and Supplementary Fig. S12). These results suggested that IL-1β exerted a major role in endothelial CD38 upregulation.

Given that macrophage infiltration-related inflammation has been shown to exacerbate hypertension and vascular dysfunction, we examined whether the pro-inflammatory macrophages were involved in the IL-1β secretion and the upregulation of CD38 in ECs. IF assay was performed with the macrophage-specific marker CD68 in the aortas of hypertensive patients and healthy subjects, and the data proved that CD68^+^ macrophages were more abundant in hypertension than in normal conditions (Supplementary Fig. S13a, b). IF staining of F4/80 in hypertensive mice demonstrated that the proportion of macrophage infiltration was much higher than that in control mice (Supplementary Fig. S13c, d). Additionally, flow-cytometric analysis of aortas from hypertensive mice showed increased macrophage accumulation compared with that in control mice (Fig. 5f).

Next, we polarized naive (M0) macrophages in the PBMCs of hypertensive patients and healthy subjects to classical pro-inflammatory macrophages and anti-inflammatory macrophages. We first determined that the IL-1β level in the cell culture supernatant of pro-inflammatory macrophages derived from hypertensive patients was higher than that in the other groups (Supplementary Fig. S14a). Then, HAECs were incubated with the cell culture supernatant of macrophages and the mRNA and protein levels of NAD^+^ consuming or biosynthetic enzymes were assessed. We found that endothelial CD38 expression was upregulated in pro-inflammatory macrophages (both LPS polarized macrophages and IFN-γ polarized macrophages) incubation (Supplementary Figs. S14b–d and S15a). In addition, protein levels were assessed by western blotting and supported the results (Fig. 5g and Supplementary Fig. S15b, c). Furthermore, we used a transwell coculture system with macrophages in the upper chamber and HAECs in the lower chamber to validate the macrophage-mediated inflammatory effect on CD38 in ECs (Supplementary Fig. S16a). A similar increase in CD38 expression was observed in cocultures of pro-inflammatory macrophages from hypertensive patients (Supplementary Fig. S16b, c). Moreover, endothelial cells, which were cocultured with macrophages transfected with IL-1β siRNA, showed lower levels of CD38 (Supplementary Fig. S17). The results further demonstrated that macrophage-derived IL-1β promoted CD38 expression. To determine whether macrophages from hypertensive patients influenced, in part, endothelial NAD^+^ levels, we investigated endothelial NAD^+^ levels in different macrophage cocultures. After 2 days, NAD^+^ levels in ECs were quantified by HPLC-MS, and there was a decrease in cellular NAD^+^ content (Fig. 5h). These observations suggested that macrophages may influence endothelial CD38 and NAD^+^ levels.

We subsequently explored how IL-1β triggered CD38 expression in ECs. The JAK/STAT pathway is an emerging target in inflammation, mainly in disorders that heighten cardiovascular risk.^31^ More importantly, the JAK/STAT pathway was identified as a modulator of CD38 expression.^26^ Thus, we examined the impact of IL-1β on the JAK1/STAT1 pathway and CD38. p-JAK1/JAK1 expression was dose-dependently upregulated by IL-1β (Fig. 5i). Additionally, the protein level of STAT1 and the phosphorylation ratio of STAT1 were elevated after IL-1β treatment, which was reversed by JAK1 inhibitor (Fig. 5i and Supplementary Fig. S18). Next, we found that the JAK1 and STAT1 inhibitors, filgotinib, and fludarabine reversed IL-1β-induced CD38 increase (Fig. 5j and Supplementary Fig. S18). To further explore the regulatory mechanism of JAK1/STAT1 pathway and CD38, a luciferase reporter assay was performed to confirm the possibility of interaction between STAT1 and CD38 promoter. Our results showed that STAT1 directly targeted the CD38 promoter region (Fig. 5k), suggesting a role for the JAK1/STAT1 pathway in CD38 expression. Taken together, these data demonstrated that the IL-1β/JAK1/STAT1 signaling pathway promoted CD38 transcription in endothelial cells.

### CD38 depletion reduces BP and improves vascular dysfunction in hypertensive mice

**Fig. 6:**
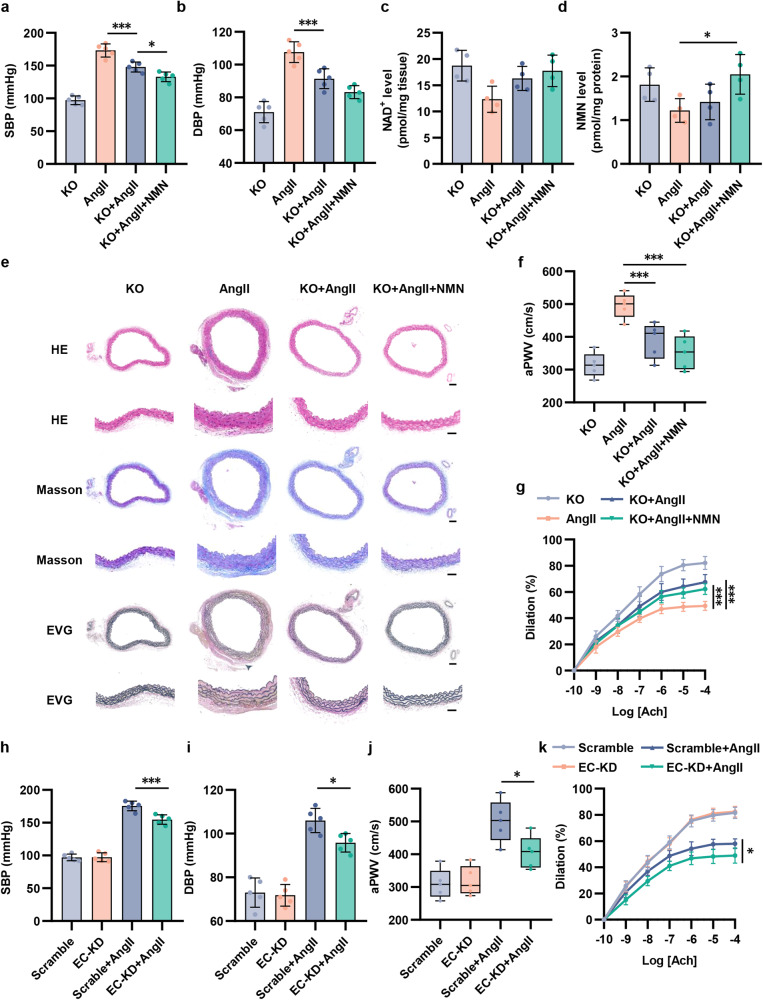
CD38 knockout and AAV-mediated endothelial CD38 knockdown reduced BP and improved vascular function in AngII-induced hypertensive mice. **a**, **b** The change of SBP and DBP were detected by a tail-cuff plethysmography system (*n* = 5). **c**, **d** NAD^+^ and NMN levels in aortas were detected by HPLC-MS (*n* = 5). **e** Representative images of aorta sections stained with HE, Masson trichrome blue and EVG staining. Scale bar (original) = 100 μm. Scale bar (magnified) = 50 μm. **f** The aortic ring assay was performed (n = 5) to evaluate the vasodilation (*n* = 5). The differences were compared to AngII group. **g** In vivo aortic pulse wave velocity was measured by Doppler (*n* = 5). **h**, **i** The change of systolic blood pressure (SBP) and diastolic blood pressure (DBP) were detected by a tail-cuff plethysmography system (*n* = 5). **j** The aortic ring assay was performed to evaluate the vasodilation (*n* = 5). **k** In vivo aortic pulse wave velocity was measured by Doppler (*n* = 5). The differences were analyzed between Scramble+AngII group and EC-KD+AngII group. *p < 0.05, ***p < 0.001

To further reveal the role of CD38 in hypertension in vivo, we used 2 strategies, including using CD38 knockout (KO) mice and CD38 inhibitors in AngII-induced hypertensive mice (Fig. 6a and Supplementary Fig. S19). As shown in Fig. 6b, c, the elevation of SBP and DBP by AngII infusion was significantly reversed in CD38 KO mice. Interestingly, the SBP of hypertensive CD38 KO mice supplemented with NMN further decreased compared with hypertensive CD38 KO mice (Fig. 6b, c), indicating that CD38 may serve as an obstacle to the function of NMN. LC–MS analyses also showed that NAD^+^ and NMN levels were significantly elevated in hypertensive CD38 KO mice (Fig. 6d, e). To evaluate the effects of CD38 on vascular remodeling, we carried out HE, Masson, and EVG staining to visualize alterations in the vasculature. Hypertensive CD38 KO mice had lower aorta media thickness and media:lumen ratio (Fig. 6f–h). However, there was no difference of that between hypertensive CD38 KO mice supplemented with or without NMN (Fig. 6f–h). Moreover, hypertensive CD38 KO mice displayed preferable aortic stiffness and endothelial-dependent vasodilation (Fig. 6i, j). Other than CD38 KO mice, 78c and isatuximab were also used to study the role of CD38. Similar to CD38 KO mice, 78c and isatuximab had the same effect in lowering BP, alleviating vascular remodeling, and improving endothelial function (Supplementary Fig. S19). To further determine the potential role of endothelial CD38 in hypertension, we carried out an adeno-associated virus (AAV)-mediated endothelial CD38 knockdown experiment in vivo (Supplementary Fig. S20a). As expected, endothelial CD38 knockdown in hypertensive mice also lowered BP, reduced aortic stiffness, and improved endothelial-dependent vasodilation (Fig. 6k–p and Supplementary Fig. S20b).

Furthermore, based on the crucial role of immune cell CD38 in regulating tissue NAD^+^ homeostasis, we performed bone marrow transplant experiments (Supplementary Fig. S21). CD38 KO mice were transplanted with bone marrow cells from wide-type (WT) mice (WT > KO) while WT mice were transplanted with bone marrow cells from KO or WT mice (KO > WT or WT > WT) (Supplementary Fig. S21a). AngII-induced hypertension mouse models were constructed based on chimera mice. Surprisingly, we found that BP (Supplementary Fig. S21b–e) and aortic structure (Supplementary Fig. S21f–h) in KO recipient mice (WT > KO) were restored compared to those in WT recipient mice (KO > WT or WT > WT). More importantly, the NAD^+^ levels in KO recipient mice were lower than that in WT recipient mice (Supplementary Fig. S21i), indicating that the hypertensive reduction in NAD^+^ and alterations in BP and aortic structure may be attributed to CD38 in endothelial cells instead of bone marrow-derived immunocytes. In addition, no differences of NAD^+^ levels (Supplementary Fig. S21i) between WT mice and WT recipient mice (KO > WT or WT > WT) were observed, suggesting that the vascular CD38 instead of immunocyte CD38 contributed to NAD^+^ reduction related BP elevation and vascular damage in hypertension.

## Discussion

**Fig. 7:**
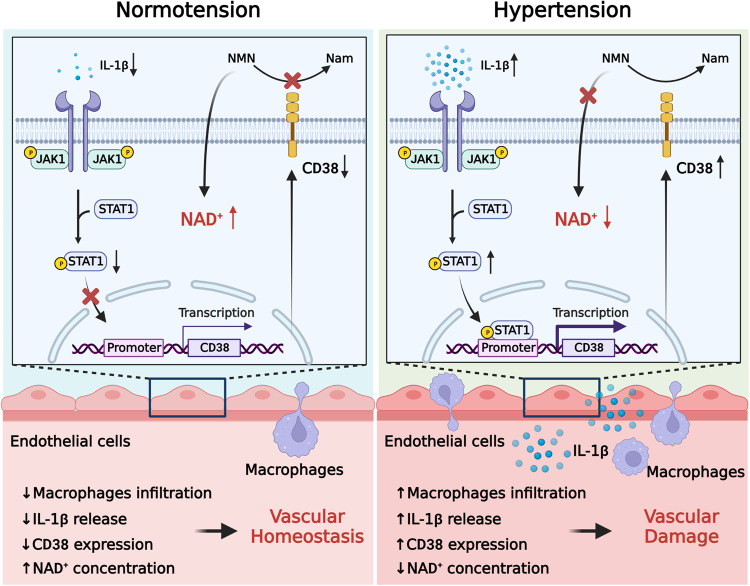
Schematic illustration. A schematic illustration showing that NAD^+^ augmentation lowered BP and improved endothelial dysfunction in the context of hypertension and showed its potential mechanisms. The illustration figure was created with BioRender.com

Our present study provides the first evidence that decline in NAD^+^ levels mediated by CD38 activation in endothelial cells plays a pivotal role in BP elevation and vascular damage in hypertensive patients, which is accompanied by enhanced infiltration of pro-inflammatory macrophages in the arterial wall. Moreover, we conducted the first NMN clinical study in hypertensive patients and showed that NMN supplement could lower BP and improve vascular function. Mechanistically, IL-1β derived from pro-inflammatory macrophages facilitates CD38 expression by activating JAK1-STAT1 signaling pathway (Fig. 7). CD38 knockout or CD38 inhibitor treatment reduces BP and ameliorates vascular dysfunction in AngII-induced hypertensive mice. To the best of our knowledge, our current data highlight that maintaining normal NAD^+^ concentration is essential for the control of BP and vascular function in patients with hypertension. NAD^+^ boosting can be used as a promising novel therapeutic strategy for the management of hypertension.

Hypertension is characterized by endothelial dysfunction and arterial stiffness, which increase the risk of cardiovascular diseases and all-cause morbidity and mortality worldwide.^4,6,30–36^ NAD^+^ is an essential cofactor in all living cells that is involved in fundamental biological processes. Studies showed that NAD^+^ deficiency has been associated with hallmarks of human health and diseases.^30,37,38^ Therefore, to evaluate whether NAD^+^ levels decline in hypertensive patients, we first measured NAD^+^ concentration in the PBMCs and found that NAD^+^ levels were significantly reduced in hypertension compared with normotension. Interestingly, the NAD^+^ levels in PBMCs is a well-acknowledged and commonly used assessment in research and in clinic,^18,39^ and its decline has been linked to the development and progression of aging and aging-related diseases, including hypertension, which was further demonstrated in our study. Furthermore, we demonstrated a systemic decrease in NAD^+^ levels not only in PBMCs but also in the aortas of hypertensive patients for the first time. The vascular wall, especially the vascular endothelium, has been generally accepted as a key contributor to the control of BP and vascular function.^2,34^ Since the NAD^+^ levels were found to be decreased in the aortas in hypertension, we next specifically focused on the NAD^+^ levels in endothelial cells to distinguish whether the alteration in endothelial NAD^+^ concentration was connected to hypertension. Both in vitro AngII-treated HAECs and MAECs isolated from AngII-induced hypertensive mice showed lower NAD^+^ levels. To further prove the beneficial effect of NAD^+^ supplement on hypertension and its vascular dysfunction, we conducted a prospective, randomized, open, 2-arm parallel, reverse translational study. NMN administration for 6 weeks in hypertensive patients, indeed, increased NAD^+^ levels in PBMCs, reduced BP and improved vascular function, which was consistent with a previous study.^18^ Additionally, NAD^+^ administration in hypertensive mice had the similar effect on BP and vascular dysfunction. A previous study also showed that NAD^+^ supplement exerted numerous beneficial effects on endothelial cells.^40^ Based on the above in vivo and in vitro studies, our findings here demonstrate for the first time that endothelial NAD^+^ deficiency contributed to BP elevation and vascular dysfunction. However, the molecular mechanisms underlying the decrease in endothelial NAD^+^ levels are still unknown.

Notably, NAD^+^ levels are determined by the dynamic balance of synthesis and consumption.^41^ CD38, which is a rate-limiting enzyme in mammalian NAD^+^ biosynthesis,^22,42^ has been reported to be highly expressed in aging and aging-related diseases.^43^ In the present study, increased CD38 expression was detected in the aortas of hypertensive patients and hypertensive mice. CD38 KO mice infused with AngII showed lower SBP, elevated NMN/NAD^+^ levels, and improved adverse vascular remodeling compared to AngII-induced hypertensive mice. Considering that several cells express CD38 including immune cells, a bone marrow transplantation experiment in CD38 KO mice was carried out to determine whether immune cells were vital for NAD^+^ consumption and BP regulation. Interestingly, only CD38 KO mice with WT bone marrow transplants showed a decrease in BP, indicating that the loss of CD38 in immune cells was not the primary factor. Inherent cells of vascular wall mainly consist of endothelial cells, vascular smooth muscle cells (VSMCs) and fibroblasts. A recent study demonstrated that CD38 of VSMCs might also contribute to BP reduction through senescence-associated small extracellular vesicles,^43^ nevertheless, given that the endothelium is the layer closest to the blood flow and endothelial function is closely related to NAD^+^ levels, we then focused on CD38 in endothelial cells instead of VSMCs. To demonstrate this issue, AAV-mediated endothelial CD38 knockdown in vivo was used to further support the results that endothelial CD38 activation contributed to hypertension and vascular dysfunction. Moreover, the in vitro experiments demonstrated that CD38 was markedly upregulated in HAEC treated with AngII and that CD38 overexpression in HAECs led to a significant decrease in NAD^+^, indicating that endothelial CD38 activation is responsible for NAD^+^ deficiency. However, the role of CD38 in VSMCs needs to be further studied in the future. Interestingly, there are 3 forms of CD38 (type II transmembrane, type III transmembrane, and soluble forms), of which the type II membrane orientation with an ecto-NAD^+^ glycohydrolase site outside the cells is the major one.^9^ In our study, AngII-induced hypertensive mice were treated with the CD38 inhibitor 78c or isatuximab, which suppressed all activities or the ecto-enzymatic activity of CD38, respectively, and were able to lower BP and reverse adverse vascular remodeling. Interestingly, a previous study used antibody 68, which was a heavy-chain antibody and a potent non-competitive inhibitor of CD38 hydrolase activity, and found that its ecto-enzymatic activity decreases levels of NAD^+^.^10^ Collectively, these data show a key role of CD38 in mediating NAD^+^ deprivation and consequently vascular dysfunction in hypertension.

Last, we assessed the potential reason for CD38 activation with a subsequent decline in NAD^+^ levels. Hypertension is a special kind of chronic inflammatory disease, and evidence indicates that the activation of endothelial inflammation promotes hypertension development.^25,34^ Thus, we explored whether inflammation activation was related to CD38 upregulation. A series of inflammatory factors were screened, and IL-1β was identified as the main regulator of CD38 expression in ECs, which was similar to a previous study.^26^ Considering that IL-1β is the primary cytokine in macrophages,^44^ we further demonstrated that infiltrated macrophages and IL-1β levels were dramatically increased in the hypertensive vessels of both humans and mice. Macrophages are considered as key players in modulating the inflammatory status in hypertension, and studies have reported that perivascular inflammation in hypertension is triggered by infiltrated macrophages.^25,45,46^ Moreover, depleting macrophages in mice attenuated hypertension, endothelial dysfunction, and vascular oxidative stress in response to AngII stimulation,^47^ which strengthened evidence of the important role of macrophages. Then, we found that IL-1β promoted JAK1/STAT1 pathway, which is a major signaling pathway in inflammation,^48^ and subsequently contributed to the enhanced CD38 expression in ECs, while inhibiting JAK1/STAT1 pathway attenuated the effect of IL-1β on CD38, suggesting that IL-1β secretion from macrophages mediated CD38 upregulation in ECs via JAK1/STAT1 pathway.

The present study has important clinical implications for hypertension. First, recent studies have demonstrated that vascular aging occurs earlier than hypertension and predicts the development of hypertension, while hypertension accelerates vascular aging.^49–51^ From the perspective of primary prevention of hypertension, vascular aging-targeted intervention may be a promising clinical strategy to slow the occurrence of hypertension. Second, accumulating evidence shows that vascular aging is emerging as a significant target for the management of cardiovascular residual risk in hypertensive patients.^4,52,53^ Thus, NAD^+^ supplement is an innovative method for reducing cardiovascular residual risk in hypertension as evidenced in our current study. Nevertheless, efforts still need to be made in the clinical study with a larger sample size to determine the clinical efficacy of NAD^+^ targeting vascular aging hallmarks. To the best of our knowledge, our study integrates the concept of delaying vascular aging into hypertension prevention and treatment, and spotlights NAD^+^ supplement as a new therapeutic approach.

In summary, the present study demonstrates that NAD^+^ augmentation lowered BP and improved vascular dysfunction in the context of hypertension, and provides robust evidence to support vascular aging-targeted intervention as an alternative and novel means to control BP and improve vascular function in hypertension. Of particular importance, we make the effort to explore the potential possibility of NAD^+^ treatment from the perspective of aging to clinical application for the management of hypertension. Overall, the present study may shed new light on novel strategies in which NAD^+^ boosting therapy, including NMN supplement and CD38 inhibition, may turn out to be a promising therapeutic measure to treat patients with hypertension.

## Materials and methods

### Ethics approval statements

All animal protocols were approved by the Animal Care and Use Committees of The First Affiliated Hospital of Sun Yat-sen University (Guangzhou, China). The clinical trial protocol and sample collection were approved by the Ethics Committee of the First Affiliated Hospital of Sun Yat-sen University. All participants or legal representatives provided their written informed consent. The clinical trial complied with the Declaration of Helsinki, and was registered at clinicaltrials.gov (NCT04903210).

### Clinical study subjects

In the cross-section study, participants from 18 to 80 years initially diagnosed with essential hypertension or on antihypertensive treatments were included in the study. Patients were excluded if they had secondary hypertension, diabetes mellitus, acute cardiovascular diseases (atrial fibrillation, myocardial infarction, unstable angina, heart failure, and stroke), peripheral vascular disease, acute or chronic liver disease, renal insufficiency, malignancies, infectious disease, using non-steroidal anti-inflammatory drugs, steroids or vasoactive agents, were known allergic to niacin or nicotinamide, received certain concurrent supplements, were currently pregnant or wished to become pregnant over the course of the study follow-up or were at suckling period.

In the clinical trial, considering the potential influence of antihypertensive therapy and the risks of not taking antihypertensive drugs for severe hypertension, we enrolled participants from 18 to 80 yrs initially diagnosed with mild essential hypertension (blood pressure ranged from 130/80 to 159/99 mmHg) in the study. The exclusion criteria of the clinical trial are consistent with those of cross-section study.

### Clinical study design, randomization, and intervention

The present prospective, randomized, open, 2-arm parallel interventional study was carried out at the First Affiliated Hospital of Sun Yat-sen University between June 2021 and March 2022. This trial complied with the Declaration of Helsinki, and was registered at clinicaltrials.gov (NCT04903210). The protocol was approved by the Ethics Committee of the First Affiliated Hospital of Sun Yat-sen University ([2021]201-2). All participants provided their written informed consent.

In total, 21 patients were enrolled and randomly assigned to the NMN (NMN10000 WRIGHT LIFE®) or lifestyle modification group for 30-day treatment using a computer-generated random list before the initiation of the trial. Patients in NMN group received 800 mg NMN once daily and instructions about lifestyle modification (intake 1400–1600 kcal/day: 54% carbohydrates, 24% proteins, 22% lipids, 108 mg cholesterol, 35 g fiber; at a low-salt diet ( < 6 g per day), avoid smoking and alcohol consumption; performing aerobic activity 4 days per week such as 45 min on a stationary bicycle), as reported in our previous study,^54^ whereas patients in the lifestyle modification group only improved their life styles. All the enrolled patients were confirmed whether they have followed the lifestyle instruction when finishing the trial. Researchers performing specimen collection and data analysis were blinded to the treatment condition.

### Outcomes and laboratory measurements

The primary outcome was the group differences of vascular function including FMD and baPWV before and after NMN treatment. The secondary outcomes were the group differences of PBMCs NAD^+^ level, blood pressure, body weight, issues with tolerability or treatment-emergent adverse events (AEs) before and after NMN treatment. Moreover, plasma inflammatory cytokines were also compared between two groups using ELISA. Body mass and BMI were recorded by anthropometry. Fasting serum glucose, serum creatinine, uric acid, aspartate aminotransferase (AST), alanine aminotransferase (ALT), triglyceride and total, LDL, and HDL cholesterol levels were measured using standardized assays at the First Affiliated Hospital of Sun Yat-sen University Laboratory at baseline and after intervention of the study.

### Animal models

All animal protocols were approved by the Animal Care and Use Committees of The First Affiliated Hospital of Sun Yat-sen University (Guangzhou, China). Cd38 KO mice were generated by Cyagen Biosciences Inc. (Guangzhou, China). In all, 8–10-week-old male CD38 KO mice and age and genetically matched WT were randomly assigned to the sham or Ang II-treatment group and were infused with saline or Ang II (Sigma-Aldrich, 0.8 mg/kg/day via subcutaneously implanted osmotic pumps for 4 weeks), respectively. CD38 inhibitor 78c (MCE, intravenous injection twice daily, 10 mg/kg/dose), isatuximab (MCE, intravenous injection twice a week, 10 mg/kg/dose), and NMN (Sigma-Aldrich, oral gavage, 300 mg/kg) were administered to WT mice over a period of 4 weeks along with Ang II infusion. Blood pressure of mice was analyzed with a computerized, non-invasive tail-cuff plethysmography system (Softron BP-2010A Blood Pressure Analysis System).

### Bone marrow transplant

Three- to four-month-old WT or CD38 KO recipient mice were irradiated with two dosages of 5 Gray (Gy) radiation 24 h apart. Bone marrow cells were isolated from tibias and femurs of 6–8-week-old WT or CD38 KO donor mice. Subsequently, 2 × 10^6^ bone marrow cells were injected retro-orbitally into irradiated recipient mice. Sulfamethoxazole (MCE, 95 mg kg^−1^ per 24 h) was dissolved in drinking water for transplant mice from 3 days before the irradiation to 2 weeks after transplant. The expression of CD38 in peripheral blood was confirmed by flow cytometry. Ten weeks after transplant, the recipient mice were treated with Ang II mini-pump and subsequent experiments.

### In vivo endothelial-specific CD38 knockdown

Scrambled siRNA and siRNA targeting mouse CD38 (siRNA: 5’-CCAAGAACCCUUGCAACAUTT-3’) were cloned into TIEp-EGFP-MIR155(MCS)-WPRE-SV40-PolyA adeno-associated virus serotype 9 (AAV9) vector (Genechem, China). Eight-week-old male C57BL/6J mice were injected with AAV9 (5.0 × 10^11 ^v.g) via tail vein, as previously described.^55^ One week later, the AAV2-injected mice were treated with were subcutaneously infused with Ang II via Alzet osmotic mini pumps.^56^ Tail-cuff blood pressure was measured. The mice were sacrificed after 28 days and aortic tissues were isolated for further morphological analysis.

### Statistical analysis

Continuous variables were reported as the means ± standard deviation (SD) while categorical variables were shown as *n* (%). Statistical significance of differences between two groups were analyzed using two-sample or paired Student’s *t* test for continuous variables and *χ*^2^ test for categorical variables. One-way analysis of variance (ANOVA) followed by Tukey’s and Dunnett’s (if only compared to the control group) multiple comparison tests were performed for multiple groups. Two-way ANOVA was adopted for the analysis in the time-dependent or concentration-dependent experiments. The Pearson’s correlation coefficient was used for correlation. All *p* values < 0.05 were considered statistically significant. Analyses were performed using with GraphPad Prism 8.

### Supplementary Information

Supplementary Materials

original western blots

SUPPLEMENTAL MATERIAL-Flow cytometry gate strategy

## Data Availability

All data supported the paper are presented in the paper and/or the Supplementary Materials. The data will be made available to other researchers with reasonable requests.
