# Supplementary material for: NAD+ exhaustion by CD38 upregulation contributes to blood pressure elevation and vascular damage in hypertension
Source: Signal Transduct Target Ther. 2023 Sep 18;8:353. doi: 10.1038/s41392-023-01577-3 (PMC10505611; doi:10.1038/s41392-023-01577-3)
Supplement: Supplementary file 1 — Supplementary Materials [file 41392_2023_1577_MOESM1_ESM.docx]

Supplementary Materials for

NAD^+^ exhaustion by CD38 upregulation contributes to blood pressure elevation and vascular damage in hypertension

Yumin Qiu^1,2,3^, Shiyue Xu^1,2,3^, Xi Chen^1,2,3^, Xing Wu^1,2,3^, Zhe Zhou^1,2,3^, Jianning Zhang^1,2,3^, Qiang Tu^1,2,3^, Bing Dong^1,2,3^, Zhefu Liu^1,2,3^, Jiang He^1,2,3^, Xiaoyu Zhang^1,2,3^, Shuangshuang Liu^1,2,3^, Chen Su^1,2,3^, Hui Huang^*,4^, Wenhao Xia^*,1,2,3,5^, Jun Tao^*,1,2,3^

Correspondence to: taojungz123@163.com; xwhzsyy@163.com; huangh8@mail.sysu.edu.cn

**This PDF file includes:**

Materials and Methods

Figures. S1 to S23

Tables S1 to S4

Materials and Methods

**Assessment of Flow-mediated dilation (FMD)**

FMD was measured by high-resolution ultrasonography equipment (UNEXEF18G, UNEX Co., Nagoya, Japan) as previously reported.^1^ After the ultrasound probe found the right brachial artery, its diameter was automatically measured and defined as baseline diameter. Subsequently, the blood pressure cuff on the right arm was inflated 50 mmHg greater than the systolic blood pressure to block the arterial blood flow for 5 minutes. Maximum vessel diameter within 1 minute after cuff release was defined as peak diameter. Percentage of FMD [FMD% = (peak diameter—baseline diameter)/baseline diameter] was applied in further analysis.

**Measurement of branchial-ankle pulse wave velocity (baPWV)**

Arterial stiffness was measured by baPWV using a noninvasive and automatic device (Omron BP-203RPEIII automatic waveform analyzer, Colin, Co, Komaki, Japan) as previously described by our team.^1^ The maximum baPWV of the left and right sides was used in the analysis.

**Human tissue samples**

Samples were collected in accordance with human research protocol approved by the Ethics Committee of the First Affiliated Hospital of Sun Yat-sen University (Guangzhou, China). Written informed consent was obtained by the organ donors’ legal representatives. All experiments used with human tissue samples were approved by the Ethics Committee and performed in accordance with the relevant guidelines. Control aortic tissue samples were obtained from heart or kidney or liver donors who suffered from traffic accident and head trauma, and diseased aortic tissue samples were obtained from donors who were diagnosed as hypertensive cerebral hemorrhage with systolic blood pressure exceeding 180 mmHg in the emergency department. Patients were excluded if they had aortic dissection, a heritable form of aortopathy (eg, Marfan syndrome), diabetes, tumor or other diseases. The donors of the two groups are age-matched. The descending aortic tissues were obtained in the transplant operation. Five samples from normal-hypertension and 5 samples from hypertension were used in the study. For each sample, a piece of aortic tissue (1-2 cm^2^) was separated and preserved in liquid nitrogen immediately in preparation for the following experiments.

**Human PBMCs isolation and macrophage experiment**

PBMCs were purified from healthy and hypertensive subjects using lymphocyte separation medium and density-gradient centrifugation. To obtain the monocytes, PBMCs (2×10^6^) were seeded into T25 flasks and incubated in a 5% CO2 incubator at 37 °C for 2–3 h with RPMI. Non-adherent cells were removed with PBS. To generate human macrophages, the adherent monocytes were cultured with 100 ng ml^-1^ recombinant human M-CSF for 7 days. Macrophages were then stimulated with 100 ng ml^-1^ LPS or 20 ng ml^-1^ IFN-γ, or 10 ng ml^-1^ recombinant human IL-4 for 1 day, in order to study the effect of pro-inflammatory macrophages and anti-inflammatory macrophages, respectively.

**Primary mouse aortic endothelial cell culture**

Primary mouse aortic endothelial cells (MAECs) were isolated and cultured from CD38 KO mice and WT mice as previous reported.^2^ Briefly, growth factor-reduced matrixgel (Corning) was completely thawed at 4 °C overnight. The precooled 35mm dish was coated with 1 mL of matrixgel and placed in a 37 °C incubator for 20 min to allow the matrixgel to solidify. The mice thoracic aorta segments were isolated and seeded lumen-side-down onto the matrixgel in ECM supplemented with endothelial cell growth factors, 10% FBS, and 1% Penicillin/Streptomycin. On the 4th day, the aortic segments were gently removed from the matrixgel without interrupting the growing endothelial cells. After proliferation for 3-4 days, the MAECs were passaged with trypsin and reseeded on 0.1% gelatin-coated cell culture plates. The MAECs were characterized by flow cytometry.

***In vivo* aortic pulse wave velocity**

Aortic pulse wave velocity (aPWV) was performed as previous description.^3^ The mice were anesthetized with 2% isoflurane in supine position with their legs fixed on ECG electrodes. Doppler probe was placed on transverse aortic arch and abdominal aorta after mice fur were shaved off. The pre-ejection time of each part was set as the time between the R-wave of the ECG to foot of the Doppler signal. aPWV was calculated by dividing the distance between the transverse and abdominal probes by the difference in their pre-ejection times.

**Aortic ring assay**

The aortic ring assay was carried out as described.^4^ Briefly, 8-week-old male mice were anaesthetized with isofluorane and the descending aorta was isolated. Mice aortic rings of 0.5-1 mm in width were cultured in collagen gel (1.5 mg/ml). The aortic rings were stimulated with NMN, 78c or isatuximab, or transfected with CD38 siRNA or CD38 lentivirus. The rings were cultured for 9 days at 37°C and 5% CO2 and the growth medium were changed every other day. The rings were photographed with an inverted microscope and the number of sprouts in each condition was quantified.

**Enzyme linked immunosorbent assay (ELISA)**

IL-1β, IL-6 and IL-18 were measured in the plasma isolated from human and mice following the manufacturer’s protocol (RayBiotech).

**NO detection**

NO production was determined by detecting the nitrite level using Griess reagent (Beyotime, Shanghai, China) according to the manufacturer's instructions. The production of NO in medium was measured at an absorbance of 405 nm.

**Cell culture**

Human aortic endothelial cells (HAECs) and MAECs were cultured in endothelial cell medium (ECM, ScienCell), supplemented with 10% fetal bovine serum (Gibco), and 1% penicillin–streptomycin (ThermoFisher, 15070063). HAECs were stimulated with Ang II at a final concentration of 10^-6^ mol L^-1^ in culture medium for 48h in the following study.^5^ Human PBMCs (2×10^6^) were cultured with complete RPMI supplemented with 10% fetal bovine serum, 1% penicillin–streptomycin, 1 mM sodium pyruvate (Glibo), 2 mM l-glutamine (Glibo), 10 mM HEPES buffer (Corning, 25-060-CI) and 50 μM 2-mercaptoethanol (ThermoFisher). Human 293T cells were maintained in DMEM supplemented with 10% fetal bovine serum, and 1% penicillin–streptomycin. Medium was changed every 48 h and passaged when cells reach 80% confluency.

**Endothelial cell adhesion, migration and tube formation**

The function of endothelial cells was performed as previously described.^6^ In adhesion assay, HAEC (2×10^4^) of different groups were planted in stimulated 6-well plates coated with fibronectin (10 µg mL^-1^) for 6 h at 37 °C. After removal of nonattached cells with PBS, the adherent ECs were fixed with 4% paraformaldehyde and stained with 0.3% crystal violet. The number of adherent cells in each section was scored under a microscope.

The migration of ECs was examined with two different assays. For Wound-healing assay, a wound in different group of 6-well plate was scraped manually with a p20 pipet tip and the scrape was photographed as the initial state. After 12 h incubation at 37 °C, transmigrated cells were observed under an optical microscope and the migration ratio was calculated. For transwell assay, Boyden chamber (8 μm pore size, Corning, USA) was used. ECM medium with 10% FBS was added into the lower chamber, and subsequently, cells (2×10^4^) with different treatment were seeded in the upper chambers filled with ECM medium without FBS. After incubation at 37 °C for 6 h, cells remaining at the upper surface of the membrane were removed using a swab, while the ECs on the lower membrane surface were fixed with 4% paraformaldehyde stained with 0.3% crystal violet. The number of migrating cells through the filter was scored.

For tube formation assay, 60 μl growth factor-reduced Matrigel (Corning) per well were seeded in 96-well culture plates for 1 h at 37 °C for solidification. ECs (2×10^4^) were resuspended with ECM and different treatment and loaded on the top of the Matrigel. Cells were incubated at 37 °C for 12 h. Tube formation was observed under an inverted microscope and an average of tubules was measured using NIH Image J software.

***In vitro* aortic vasorelaxation**

*In vitro* aortic vasorelaxation assay was carried out based on our previous reports.^3^ After the force calibration of Wire Myograph System 620M (DMT620M, Denmark), the mice were sacrificed and the thoracic aorta segments were cleaned and cut into slices of 2 mm in length without stretching. The aorta rings were quickly and carefully suspended between two stainless steel wires in an organ chamber filled with Krebs solution at 37 °C with 95% O2 and 5% CO2. After an equilibration of 40 min, 1 µM phenylephrine (PE) and 30 µM acetylcholine (ACh) were added to the organ chamber for assessment of contractile activity and endothelial integrity, respectively. After washing and re-equilibration for 40 min, an incremental PE dose (from 10 nM to 10 mM) or sodium nitroprusside (SNP) (from 10nM to 10mM) was added to the organ chamber to obtain a concentration-dependent contractile curve. Vasorelaxation was expressed as percentage reversal of the phenylephrine-induced contraction. All data were analyzed with the Panlab system (Panlab Harvard Apparatus, Barcelona, Spain).

**NAD^+^, NMN and** **other metabolite measurement by HPLC-MS**

The abundance of NAD^+^ and other metabolite were detected by HPLC-MS as reported previously.^7^ Briefly, the internal standard solution was made up using Nicotinamide-^13^C_6_ solution (Sigma. V-034, 100 μg/mL in methanol). Before each batch of samples was measured, 80% methanol solution with internal standard nicotinamide concentration of 50 ng/ml was prepared and was precooled at -80 °C. For cells testing, the culture medium was changed 2 hours before measurement so that the cells were in a state of sufficient nutrition and vigorous growth. The cells were washed with 1 ml PBS at 4 °C, and subsequently, 1 ml standard solution was added into the cell on liquid nitrogen. After incubation at -80 °C for 20min, the cells were scraped and centrifuged at 4 °C at 15000g for 30min. The supernatant was collected. For tissue testing, 20-40 mg tissue was ground with zirconia grinding beads in standard solution. The tissue was centrifuged at 4 °C at 15000g for 30min and the supernatant was collected. For cell culture supernatant testing, 800 μl standard solution was mixed with 200 μl cell culture supernatant, and the mixture was gently shaken and incubated for 6–8h at − 80 °C. Then, the mixture was centrifuged at 4 °C at 15,000g for 30 min. The resulting supernatant was transferred to a new centrifuge tube and stored on ice, while the remaining pellet was reserved for protein concentration analysis using the Bradford assay.

A 1200 series HPLC from Agilent (Santa Clara, CA) was used for separation of NAD^+^ and metabolites. Buffer A consisted of 100% acetonitrile and buffer B consisted of 95:5 water with 20 mm ammonium acetate adjusted to pH 9.6 with 20 mm ammonium hydroxide. Twenty microliters of the extracted sample were analyzed at a flow rate of 400 μl per min (back pressure should not exceed ~3,000 p.s.i. at 2% (vol/vol) B) and the gradient was as follows: 0 min, 85% B; 3 min, 85-30% B; 12 min, 30-2% B; 15 min, 2% B; 16 min, 2-85% B; 23 min, 85% B. MS analysis was carried out on an Agilent 6410 triple quadrupole mass spectrometer in positive ionization mode. Calibration curves for each NAD^+^ metabolites were constructed using Agilent Masshunter Quantitative Analysis software. The concentration of NAD^+^ metabolites was quantitated with calibration curves and normalized by the protein concentration of the pellet.

**siRNA transfection**

Small-interfering RNAs (siRNAs) against CD38 or IL-1β, alone with negative control (Shanghai Obio Technology, Shanghai, China), were transfected into ECs or macrophages, respectively, using Lipofectamine 3000 (ThermoFisher, Waltham, MA), as in our prior studies.^5^ The sequences of hCD38 and hIL-1β siRNA were shown as followed:

siCD38-1: sense (5'-3') GAGAUGAGACAUGUAGACUGCCAAAdTdT and antisense (5'-3') UUUGGCAGUCUACAUGUCUCAUCUCdTdT;

siCD38-2: sense (5'-3') GCUUUCAAGGGUGCAUUUAUUdTdT and antisense (5'-3') AAUAAAUGCACCCUUGAAAGCdTdT;

siCD38-3: sense (5'-3') GGUGUGGUGAAUUCAACACUUdTdT and antisense (5'-3') AAGUGUUGAAUUCACCACACCdTdT.

siIL-1β-1: sense (5'-3') CAAAGAAGAAGAUGGAAAAdTdT and antisense (5'-3') UUUUCCAUCUUCUUCUUUGdTdT;

siIL-1β-2: sense (5'-3') GAGAAGAAAGUAAUGACAAdTdT and antisense (5'-3') UUGUCAUUACUUUCUUCUCdTdT;

siIL-1β-3: sense (5'-3') GCACCUGUACGAUCACUGAdTdT and antisense (5'-3') UCAGUGAUCGUACAGGUGCdTdT.

**Lentivirus production and infection**

Recombinant lentivirus encoding CD38 (pCMV-CD38-EGFP-3FLAG) was constructed by cloning the CD38 gene into the pCMV-EGFP-3FLAG vector (Shanghai Obio Technology, Shanghai, China). ECs were transduced with lentivirus encoding human CD38 gene (LV-CD38) or negative control gene (LV-NC) at multiplicity of infection of 20, and cells were re-fed with fresh medium after 16 hours of transfection.

**RNA Isolation and RT-PCR**

Total RNA of cells or tissues was extracted using TRIzol (Invitrogen, ThermoFisher, CA). RT-PCR was carried out as previously described.^8^ Detailed primers sequences for each gene are listed in Table S3.

**Immunoblotting**

Protein extracts of cells and tissues were obtained and immunoblotted as previously described.^8^ The antibody used are shown in Table S4.

**Flow cytometry**

ECs in different groups were stained with fluorochrome-conjugated monoclonal antibodies against CD38 for 30 min at 4 °C. The mean fluorescence intensity (MFI) of CD38 was quantified by CytoFlex S (Beckman Coulter). Relative CD38 MFI was normalized to MFI of control cells. Besides, aortas from control mice and hypertensive mice were isolated and stained with fluorochrome-conjugated monoclonal antibodies including CD45, F4/80, and CD11b (Supplemental Table 4). The corresponding isotype controls were also used for gate selection. Multicolor flow cytometry was performed using an CytoFlex S. All the data were analyzed by FlowJo V10 software (Treestar, San Carlos, California, USA).

**Luciferase assay**

293T cells were transfected with pGL4.10 vectors containing CD38 promoter, or pcDNA3.1 vectors containing STAT1, or both, together with Renilla vector with Lipofectamine 3000. pRL-TK was used for transfection control. Luciferase assay was conducted using the Luciferase Reporter Assay System (Promega, E1910). Both firefly and renilla luminous intensity was detected using a luminometer.

**Histology**

Thoracic aortas of mice with various treatment were isolated and fixed with 4% formaldehyde. After paraffin embedding, the aortas were cut into 4-μm sections, and stained with hematoxylin–eosin (H&E), Masson, Gomori’s aldehyde-fuchsin dye and Von Kossa. The luminal radius, media thickness, and intensity of collagen and elastin staining were calculated by ImageJ software, and the media-to-lumen ratio was quantified, as previous reported.^9^

**Immunofluorescence staining and confocal microscopy**

The thoracic aorta paraffin slides of the human and mice were deparaffinized and submerged in EDTA antigen retrieval buffer (pH 9.0) and microwaved for antigen retrieval. The slides were permeabilized with 0.1% Triton-100 for 5 min, blocked with 5% normal goat serum for 1h at room temperature and incubated overnight at 4 °C with anti-CD31 antibody. After washing with TBST, a secondary antibody was used. Subsequently, the slides were subjected to citric acid antigen retrieval buffer (pH 6.0) and blocked with 5% normal goat serum for 1h at room temperature. The primary antibody, including CD38, CD68, F4/80 and IL-1β antibody were used for incubation overnight at 4 °C. Then the different secondary antibodies were added. After DAPI staining, micrographs of all immunostained tissues were visualized via an inverted fluorescence microscope (Leica DMI8). The fluorescence intensity is calculated by the ratio of total optical density of fluorescence in the image divided by the total area of fluorescence.

**Enzymatic activity**

CD38 hydrolase activity was measured using CD38 Activity Assay Kit (abcam). The CD38 activity was calculated with a constructed standard curve.

**REFERENCES**

1 Zhang, J. et al. Non-invasive Systemic Hemodynamic Index in Vascular Risk Stratification. Tailored for Hypertensives. *Front. Cardiovasc. Med*. **8**, 744349 (2021).

2 Li, H. et al. Novel Role of GPR35 (G-Protein-Coupled Receptor 35) in the Regulation of. Endothelial Cell Function and Blood Pressure. *Hypertension* **78**, 816-830 (2021).

3 de Picciotto, N. E. et al. Nicotinamide mononucleotide supplementation reverses vascular. dysfunction and oxidative stress with aging in mice. *Aging Cell* **15**, 522-530 (2016).

4 Yang, Q. et al. PRKAA1/AMPKalpha1-driven glycolysis in endothelial cells exposed to. disturbed flow protects against atherosclerosis. *Nat. Commun*. **9**, 4667 (2018).

5 Guo, J. et al. Endothelial SIRT6 Is Vital to Prevent Hypertension and Associated. Cardiorenal Injury Through Targeting Nkx3.2-GATA5 Signaling. *Circ. Res*. **124**, 1448-1461 (2019).

6 Yu, B. et al. Bimodal Imaging-Visible Nanomedicine Integrating CXCR4 and VEGFa. Genes Directs Synergistic Reendothelialization of Endothelial Progenitor Cells. *Adv. Sci. (Weinh)* **7**, 2001657 (2020).

7 Yuan, M., Breitkopf, S. B., Yang, X. & Asara, J. M. A positive/negative ion-switching, targeted mass spectrometry-based metabolomics platform for bodily fluids, cells, and fresh and fixed tissue. *Nat. Protoc*. **7**, 872-881 (2012).

8 He, J. et al. Inhibition of Mitochondrial Oxidative Damage Improves Reendothelialization. Capacity of Endothelial Progenitor Cells via SIRT3 (Sirtuin 3)-Enhanced SOD2 (Superoxide Dismutase 2) Deacetylation in Hypertension. *Arterioscler. Thromb. Vasc. Biol*. **39**, 1682-1698 (2019).

9 Gan, L. et al. CD38 deficiency alleviates Ang II-induced vascular remodeling by inhibiting. small extracellular vesicle-mediated vascular smooth muscle cell senescence in mice. *Signal Transduct. Target Ther*. **6**, 223 (2021).

Figure. S1.

**Fig. S1 Hypertensive patients displayed a worse vascular function compared with healthy subjects.** **a-b.** Flow mediated dilation (FMD) (a) and branchial-ankle pulse wave velocity (baPWV) (b) were measured in hypertensive patients (*n*=50) and healthy subjects (*n*=52). ***p* < 0.01, ****p* < 0.001.

Figure. S2.

**Fig. S2 Subgroup analysis was performed in the relationship between NAD^+^ level and FMD and baPWV.** **a-b.** The correlation between NAD^+^ level and FMD in hypertensive patients (a) and healthy subjects (b). **c-d.** The correlation between NAD^+^ level and FMD in hypertensive patients (c) and healthy subjects (d).

Figure. S3.

**Fig. S3 The flow diagram of clinical study.**

Figure. S4.

**Fig. S4 The reduced level of p-eNOS was rescued by NMN supplement in AngII-treated HUVECs as well as the production of NO in medium. a-b.** Representative Western blot gel and summarized data of the relative protein levels of p-eNOS in HUVECs with different treatments (*n*=4). **c.** NO levels in the medium of HUVECs in different groups (*n*=5). ***p* < 0.01, ****p* < 0.001.

Figure. S5.

**Fig. S5 NAD^+^ level was decreased in aortas in hypertension.** **a-h.** NAD^+^ and its metabolites levels in human aortas were detected by HPLC-MS in hypertensive patients (*n*=5) and healthy subjects (*n*=5). **i.** Flowchart of metabolism of NAD^+^ and its metabolites. **p* < 0.05.

Figure. S6.

**Fig. S6 AngII-induced hypertensive mice showed vascular dysfunction.** **a-d.** The systolic blood pressure (SBP) (a-b) and the diastolic blood pressure (DBP) (c-d) of mice were measured with a non-invasive tail-cuff plethysmography system (*n*=5). **e.** The aortic ring assay was carried out in hypertensive mice and the control mice (*n*=4). **f.** *In vivo* aortic pulse wave velocity was detected by Doppler (*n*=4). ***p* < 0.01, ****p* < 0.001.

Figure. S7.

**Fig. S7 NMN supplement attenuated vascular remodeling in AngII-induced hypertensive mice. a.** Schematic illustration of experiment. **b.** Representative images of aorta sections stained with HE, Masson trichrome blue and EVG staining. Scale bar (original) = 100 μm. Scale bar (magnified) = 50 μm. **c-d.** The aortic media thickness and media-to-lumen ratio of the aortas were calculated by H&E staining (*n*=5). **e**. The aortic ring assay was performed to evaluate the SNP-vasodilation (*n*=5). **p* < 0.05, ***p* < 0.01, ****p* < 0.001.

Figure. S8.

**Fig. S8 CD38 was highly expressed in endothelial cells in hypertensive mice.** Immunofluorescence of the CD38 (red), endothelial cells marker CD31 (green) and DAPI-stained nuclei (blue) in aorta from hypertensive mice and control mice. Scale bar = 100 μm.

Figure. S9.

**Fig. S9 Validation of CD38 expression in endothelial cells transfected with siRNA and lentiviruses. a.** mRNA level of endothelial cells transfected with CD38 and negative control (NC) siRNA (*n*=3). **b.** mRNA level of endothelial cells transfected with CD38 and negative control (NC) lentiviruses (*n*=3). **c.** Western blot analysis of FLAG in endothelial cells transfected with CD38 and negative control (NC) lentiviruses (*n*=3). ****p* < 0.001.

Figure. S10.

**Fig. S10 Endothelial function changed in endothelial cells treated with NMN, CD38 siRNA, CD38 lentiviruses, 78c and isatuximab. a-e.** Quantification of wound healing (a), adhesion (b), transwell (c), tube formation (d) and aortic ring (e) assays in Fig 4e (*n*=5). The differences were compared to NMN group. **p* < 0.05, ****p* < 0.001.

Figure. S11.

**Fig. S11 Inflammation occurred in the hypertensive aorta. a.** mRNA of inflammatory factors in human aortas of healthy subjects (CTL) and hypertensive patients (HTN) (*n*=4). **b-c.** Western blot analysis of inflammatory factors in human aortas of healthy subjects (CTL) and hypertensive patients (HTN) (*n*=4). **p* < 0.05.

Figure. S12.

**Fig. S12 IL-1β was upregulated in endothelial cells in hypertensive mice. a.** Immunofluorescence of the IL-1β (red), endothelial cells marker CD31 (green) and DAPI-stained nuclei (blue) in aorta from hypertensive mice and control mice (*n*=4). Scale bar = 100 μm. **b.** Quantification of immunofluorescence tense. ****p* < 0.001.

Figure. S13.

**Fig. S13 More macrophages were infiltrated in aortas in hypertension. a-b.** Immunofluorescence of the macrophages marker CD68 (red) and DAPI-stained nuclei (blue) in aorta from hypertensive patients and healthy subjects (*n*=4). Scale bar (original) = 250 μm. Scale bar (magnified) = 25 μm. **c-d.** Immunofluorescence of the macrophages marker F4/80 (red), endothelial cells marker CD31 (green) and DAPI-stained nuclei (blue) in aorta from hypertensive mice and control mice (*n*=5). Scale bar = 100 μm. ****p* < 0.001.

Figure. S14.

**Fig. S14 The change of IL-1β concentration in macrophages** **supernatant and the alteration of NAD^+^ biosynthetic and consuming enzymes in endothelial cells coculture with macrophages** **supernatant. a.** IL-1β concentration of cell culture supernatant of M0, LPS polarized and IL-4 polarized macrophages from PBMCs of hypertensive patients and healthy subjects was detected by ELISA. **b.** Schematic of coculture model. **c.** mRNA of NAD^+^ biosynthetic enzymes in endothelial cells treated with cell culture supernatant of M0, LPS polarized and IL-4 polarized macrophages from PBMCs of hypertensive patients and healthy subjects (*n*=3). **d.** mRNA of NAD^+^ consuming enzymes in endothelial cells treated with cell culture supernatant of M0, LPS polarized and IL-4 polarized macrophages from PBMCs of hypertensive patients and healthy subjects (*n*=3). **p* < 0.05, ***p* < 0.01, ****p* < 0.001.

Figure. S15.

**Fig. S15 The mRNA and protein of CD38 in endothelial cells coculture with IFN-γ polarized and IL-4 polarized macrophages** **supernatant. a-c.** mRNA **(a)** and protein **(b-c)** level of CD38 in endothelial cells treated with cell culture supernatant of IFN-γ polarized and IL-4 polarized macrophages from PBMCs of hypertensive patients and healthy subjects (*n*=3). The differences were compared to CTL-M0 group. **p* < 0.05, ***p* < 0.01, ****p* < 0.001.

Figure. S16.

**Fig. S16 The change of NAD^+^ biosynthetic and consuming enzymes in endothelial cells coculture with macrophages. a.** Schematic of coculture model. **b.** mRNA of NAD^+^ biosynthetic enzymes in endothelial cells treated with M0, LPS polarized and IL-4 polarized macrophages from PBMCs of hypertensive patients and healthy subjects (*n*=3). **c.** mRNA of NAD^+^ consuming enzymes in endothelial cells treated with M0, LPS polarized and IL-4 polarized macrophages from PBMCs of hypertensive patients and healthy subjects (*n*=3). ****p* < 0.001.

Figure. S17.

**Fig. S17 The validation of IL-1β siRNA and the effect of macrophages IL-1β knockdown on endothelial CD38. a.** mRNA level of cells transfected with IL-1β and negative control (NC) siRNA (*n*=3). Based on the results, siIL-1β-2 was used in the following study. The differences were compared to siNC group. **b.** Western blot analysis of IL-1β cells transfected with IL-1β and negative control siRNA (*n*=3). **c.** Western blot analysis of CD38 in endothelial cells cocultured with macrophages which were transfected with IL-1β and negative control siRNA (*n*=3). **p* < 0.05, ***p* < 0.01, ****p* < 0.001.

Figure. S18.

**Fig. S18 JAK1-STAT1 signal pathway played an important role in CD38 expression. a-c.** Quantification of immunoblotting in Figure 5A (a), Figure 5I (b) and Figure 5J (c) (*n*=3). The differences were compared to CTL-M0 group (a), CTL group (b) and CTL group (c), respectively. ****p* < 0.001.

Figure. S19.

**Fig. S19 CD38 inhibitor administration lowered BP and improved vascular function in AngII-induced hypertensive mice. a.** Schematic of experiment. **b-c.** The change of systolic blood pressure (SBP) and diastolic blood pressure (DBP) were detected by a tail-cuff plethysmography system (*n*=5). **d-e.** NAD^+^ and NMN levels in aortas were detected by HPLC-MS (*n*=5). **f.** Representative images of aortic sections stained with HE, Masson trichrome blue and EVG staining. Scale bar (original) = 100 μm. Scale bar (magnified) = 50 μm. **g-h.** The aortic media thickness and media-to-lumen ratio of the aortas were calculated by H&E staining. **i.** The aortic ring assay was performed to evaluate the vasodilation (*n*=5). **j.** *In vivo* aortic pulse wave velocity was measured by Doppler (*n*=5). The differences were analyzed between AngII group and AngII+78c group or AngII+Isa group. **p* < 0.05, ***p* < 0.01, ****p* < 0.001.

Figure. S20.

**Fig. S20 CD38 knockout reduced BP and improved vascular function in AngII-induced hypertensive mice. a.** Schematic of experiment. **b-c.** The aortic media thickness and media-to-lumen ratio of the aortas were calculated by H&E staining (*n*=5). ***p* < 0.01.

Figure. S21.

**Fig. S21** **AAV mediated endothelial CD38 knockdown lowered BP and improved vascular function in AngII-induced hypertensive mice. a.** Schematic of experiment. **b.** Representative images of aortic sections stained with HE, Masson trichrome blue and EVG staining. Scale bar (original) = 100 μm. Scale bar (magnified) = 50 μm. **c-d.** The aortic media thickness and media-to-lumen ratio of the aortas were calculated by H&E staining (*n*=5). ***p* < 0.01.

Figure. S22.

**Fig. S22 The influences of lymphocytes on blood pressure in AngII-induced hypertensive CD38 KO mice by bone marrow transplantation. a.** Schematic of the bone marrow transplantation methods and the bone marrow chimera construction. WT > KO means that the bone marrow was replaced with WT donor cells in CD38 KO recipient mice. KO > WT means that the bone marrow was replaced with CD38 KO donor cells in WT recipient mice. WT > WT means that the bone marrow was replaced with WT donor cells in WT recipient mice. **b-e.** The systolic blood pressure (SBP) and diastolic blood pressure were measured in different group of mice (*n*=6). The differences were analyzed between WT > WT + AngII group and WT > KO + AngII group. **p* < 0.05, ****p* < 0.001.

Figure. S23.

**Fig. S23 The influences of lymphocytes on vascular function in AngII-induced hypertensive CD38 KO mice by bone marrow transplantation. a.** Representative images of aortic sections stained with HE, Masson trichrome blue and EVG staining. Scale bar (original) = 100 μm. Scale bar (magnified) = 50 μm. **b-c.** The aortic media thickness and media-to-lumen ratio of the aortas were calculated by H&E staining (*n*=5). **d.** NAD^+^ level in aortas were detected by HPLC-MS (*n*=5). **p* < 0.05, ***p* < 0.01, ****p* < 0.001.

Table S1. Adverse events for NMN group

| Adverse events | NMN group (*n*=9) |
| --- | --- |
| Any adverse event (n) | 0 |
| Gastrointestinal disorders (n) | 0 |
| Hypoglycemia (n) | 0 |
| AST and/or ALT ≥2 times upper normal limit (n) | 0 |
| Hyperuricemia (n) | 0 |
| Anaphylaxis (n) | 0 |

Table S2. NAD^+^ metabolites in PBMCs of study participants

| Metabolites | LM (*n*=10) | | | NMN (*n*=9) | | | *P_difference_* |
| --- | --- | --- | --- | --- | --- | --- | --- |
|  | Baseline | 6 weeks | *P* | Baseline | 6 weeks | *P* |  |
| NAD^+^ (pmol/mg protein) | 16.20±10.37 | 16.15±8.56 | 0.98 | 14.81±11.68 | 21.19±11.83 | 0.031 | 0.04 |
| NMN (pmol/mg protein) | 4.02±2.62 | 3.70±1.63 | 0.71 | 3.95±2.68 | 6.77±3.28 | 0.003 | 0.01 |
| NR (pmol/mg protein) | 0.56±0.37 | 0.57±0.43 | 0.90 | 0.53±0.32 | 0.57±0.32 | 0.41 | 0.75 |
| NADH (pmol/mg protein) | 1.16±1.06 | 1.15±0.96 | 0.96 | 1.14±0.93 | 1.30±0.83 | 0.12 | 0.24 |
| NADP^+^ (pmol/mg protein) | 12.49±10.51 | 12.29±9.65 | 0.77 | 11.22±6.94 | 12.96±10.42 | 0.27 | 0.23 |
| Nam (pmol/mg protein) | 184.30±131.22 | 177.94±120.93 | 0.43 | 171.24±118.23 | 196.22±126.39 | 0.02 | 0.14 |
| ATP (pmol/mg protein) | 2872.07±1868.89 | 3025.88±1427.39 | 0.47 | 2770.20±2243.59 | 3041.88±2407.86 | 0.06 | 0.64 |

Continuous variables are described as mean ± SD. Two-sided paired *t* test was used for the comparison within groups. *P_difference_* indicates the *P* value of differences before and after intervention between LM and NMN group.

Table S3. Primers for RT-PCT

| **Gene** | **Forward** | **Reverse** |
| --- | --- | --- |
| *CD38* | GCTCAATGGATCCCGCAGTA | ATCACCCAGGCCTCTAGTGT |
| *CD157* | CATTGGGAGTCAGACTGCTTG | ACAAGCATGCAGTGTCCGTG |
| *SARM1* | GGATTTCATGTGGGGAACCCT | TTGGGGAAGGAGGAGGTTCA |
| *PARP1* | CCCCACGACTTTGGGATGAA | AGACTGTAGGCCACCTCGAT |
| *PARP2* | TCCCCTGCCAAGAAAACTCG | GCCTTCACAGATTCATCTTGCT |
| *NAMPT* | GGATTTCATGTGGGGAACCCT | TTGGGGAAGGAGGAGGTTCA |
| *NMNAT1* | CCCAGAGGCTTGGAAAAGACA | CCACAAGCAAGGAGAACCAC |
| *NMNAT2* | CATCTGCACCATGACCGAGA | TGTGCAGATAATCCCTGGCTC |
| *NMNAT3* | CTACACCAAACAGCTGTGCC | TCCACTATTTCCTGGATGTGCG |
| *SIRT1* | TCGCAACTATACCCAGAACATAGACA | CTGTTGCAAAGGAACCATGACA |
| *SIRT2* | GAACGCTGTCGCAGAGTCATC | GGTTGGCTTGAACTGCCCAG |
| *SIRT3* | CCCCAAGCCCTTTTTCACTTT | CGACACTCTCTCAAGCCCA |
| *SIRT4* | AGCCTCCATTGGGTTATTTGTG | TCTGGTATCCCCGATTCGGT |
| *SIRT5* | TGGAGGAGGTTGACAGAGAGC | CTGCTGGGTACACCACAGA |
| *SIRT6* | CCCACGGAGTCTGGACCAT | CTCTGCCAGTTTGTCCCTG |
| *SIRT7* | AGAAGCGTTAGTGCTGCCG | GAGCCCGTCACAGTTCTGAG |
| *JAK1* | ACCACGCTCTGGGAAATCTG | CATGATGGTGTCACTGGCCT |
| *STAT1* | TCAGAGCTCGTTTGTGGTGG | CATTCACATCTCTCAACTTCACAGT |
| *IL-1β* | GCCCATCCTCTGTGACTCAT | AGGCCACAGGTATTTTGTCG |
| *IL-6* | TTCAATGAGGAGACTTGCCTG | CTGGCATTTGTGGTTGGGTC |
| *IL-18* | GCCTGTGTTCGAGGATATGACT | CCTTCACAGAGAGGGTCACAG |
| *ACTB* | GATTCCTATGTGGGCGACGA | TCTCCATGTCGTCCCAGTTG |
| *GAPDH* | GGAAGCTTGTCATCAATGGAAATC | TGATGACCCTTTTGGCTCCC |

Table S4. Antibodies used for immunoblotting, immunofluorescence and flow cytometry

| **Antibody** | **Supplier** | **Identifier** |
| --- | --- | --- |
| Rabbit Anti-Human CD38 | Abcam | Cat# ab108403 |
| Rabbit Anti-Mouse CD38 | Abcam | Cat# ab216343 |
| Rabbit Anti-Human CD38 | Affinity Biosciences | Cat# DF6551 |
| Mouse Anti-Human SIRT1 | Cell Signaling Technology | Cat# 8469 |
| Rabbit Anti-Human SIRT3 | Cell Signaling Technology | Cat# 2627 |
| Rabbit Anti-Human PARP1 | Cell Signaling Technology | Cat# 9532 |
| Rabbit Anti-Human CD157 | Proteintech | Cat# 16337-1 |
| Rabbit Anti-Human NAMPT | Affinity Biosciences | Cat# DF6059 |
| Mouse Anti-Human JAK1 | Proteintech | Cat# 66466-1 |
| Rabbit Anti-Human JAK2 | Cell Signaling Technology | Cat# 3230 |
| Rabbit Anti-Human STAT1 | Cell Signaling Technology | Cat# 14994 |
| Rabbit Anti-Human Phospho-STAT1 (Tyr701) | Affinity Biosciences | Cat# AF3300 |
| Rabbit Anti-Human p38 | Cell Signaling Technology | Cat# 8690 |
| Rabbit Flag Antibody | Proteintech | Cat# 80010-1 |
| Rabbit Anti-Human ACTB | Cell Signaling Technology | Cat# 8457 |
| Rabbit Anti-Human α-tubulin | Proteintech | Cat# 11224-1 |
| Anti-Rabbit IgG, HRP-linked antibody | Cell Signaling Technology | Cat# 7074 |
| Anti-Mouse IgG, HRP-linked antibody | Cell Signaling Technology | Cat# 7076 |
| Rabbit Anti-Human CD31 | Abcam | Cat# ab182981 |
| Rabbit Anti-Human CD68 | Abcam | Cat# ab213363 |
| Rabbit Anti-Mouse F4/80 | Cell Signaling Technology | Cat# 70076 |
| Rabbit Anti-Human IL-1β | Cell Signaling Technology | Cat# 12703 |
| Rabbit Anti-Mouse IL-1β | Abcam | Cat# ab234437 |
| Rabbit Anti-Human IL-6 | Proteintech | Cat# 21865-1 |
| Rabbit Anti-Human IL-18 | Proteintech | Cat# 10663-1 |
| Rabbit Anti-Human eNOS | Proteintech | Cat# 27120-1 |
| Rabbit Anti-Human Phospho-eNOS (Ser1177) | Affinity Biosciences | Cat# AF3247 |
| PE-labeled anti-human CD38 Antibody | BioLegend | Cat# 356604 |
| FITC-labeled anti-mouse CD45 Antibody | BioLegend | Cat# 109806 |
| PE-labeled anti-mouse F4/80 Antibody | BioLegend | Cat# 123110 |
| APC-labeled anti-mouse CD11b Antibody | BioLegend | Cat# 101212 |
