# Supplementary material for: NAD+ exhaustion by CD38 upregulation contributes to blood pressure elevation and vascular damage in hypertension
Source: Signal Transduct Target Ther. 2023 Sep 18;8:353. doi: 10.1038/s41392-023-01577-3 (PMC10505611; doi:10.1038/s41392-023-01577-3)

Figure3b

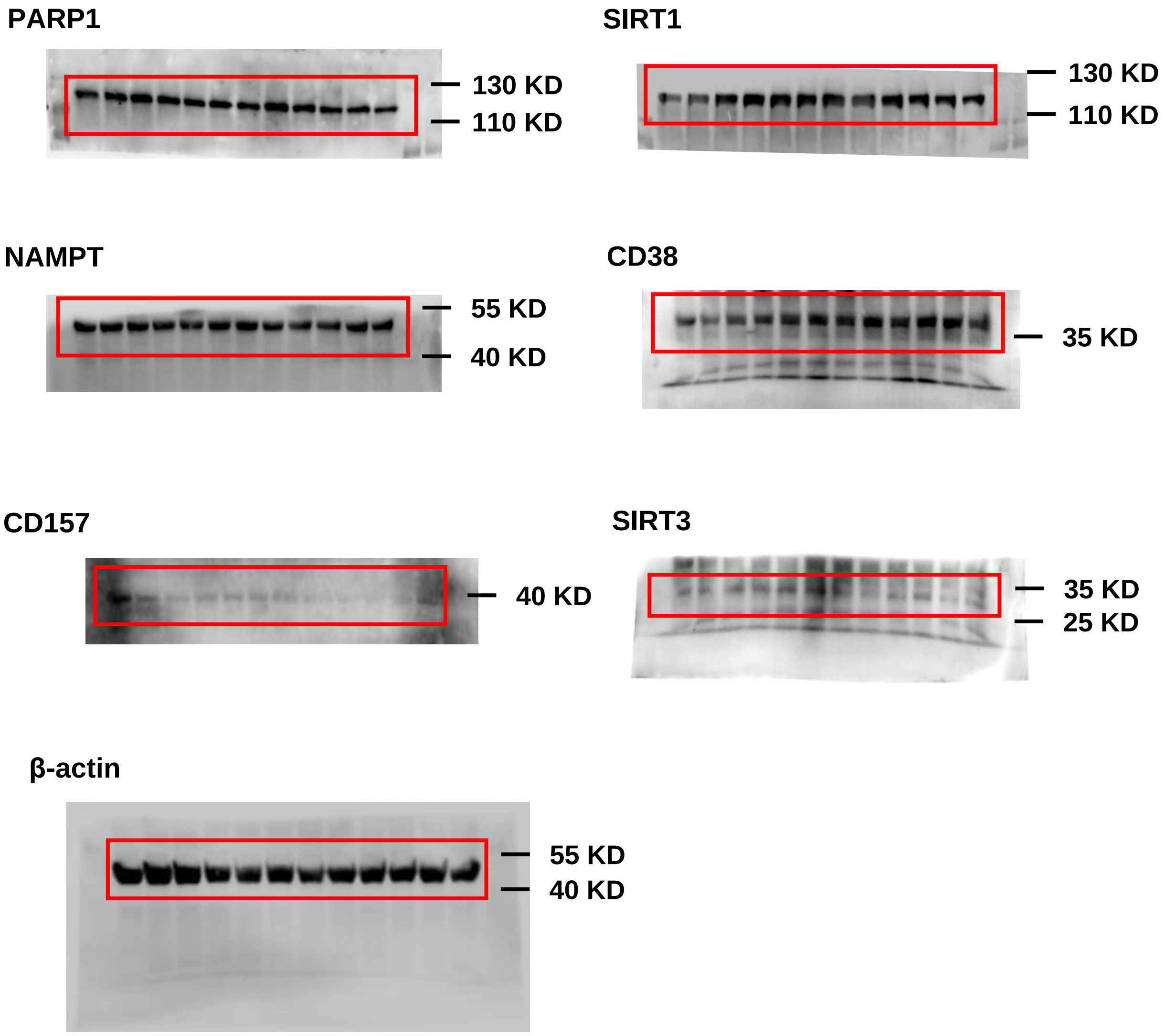
PARP1
130 KD
110 KD
SIRT1
130 KD
110 KD
NAMPT
55 KD
40 KD
CD38
35 KD
CD157
40 KD
SIRT3
35 KD
25 KD
β-actin
55 KD
40 KD

Figure 3h

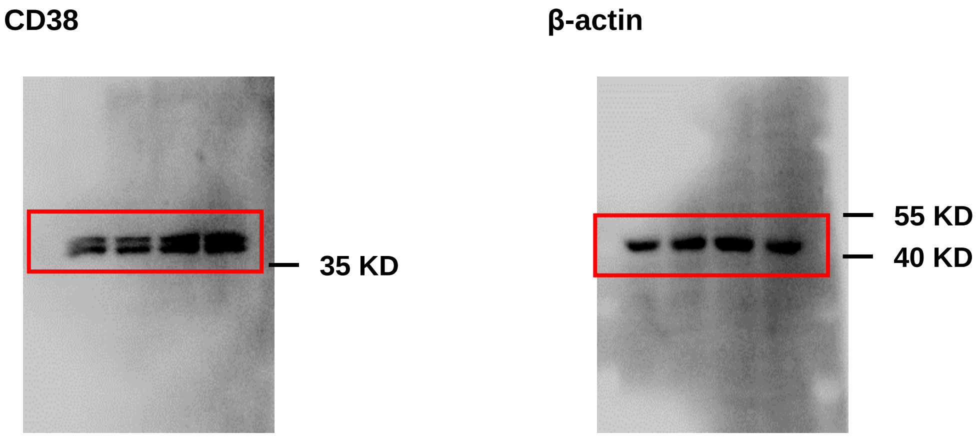

Figure 5g

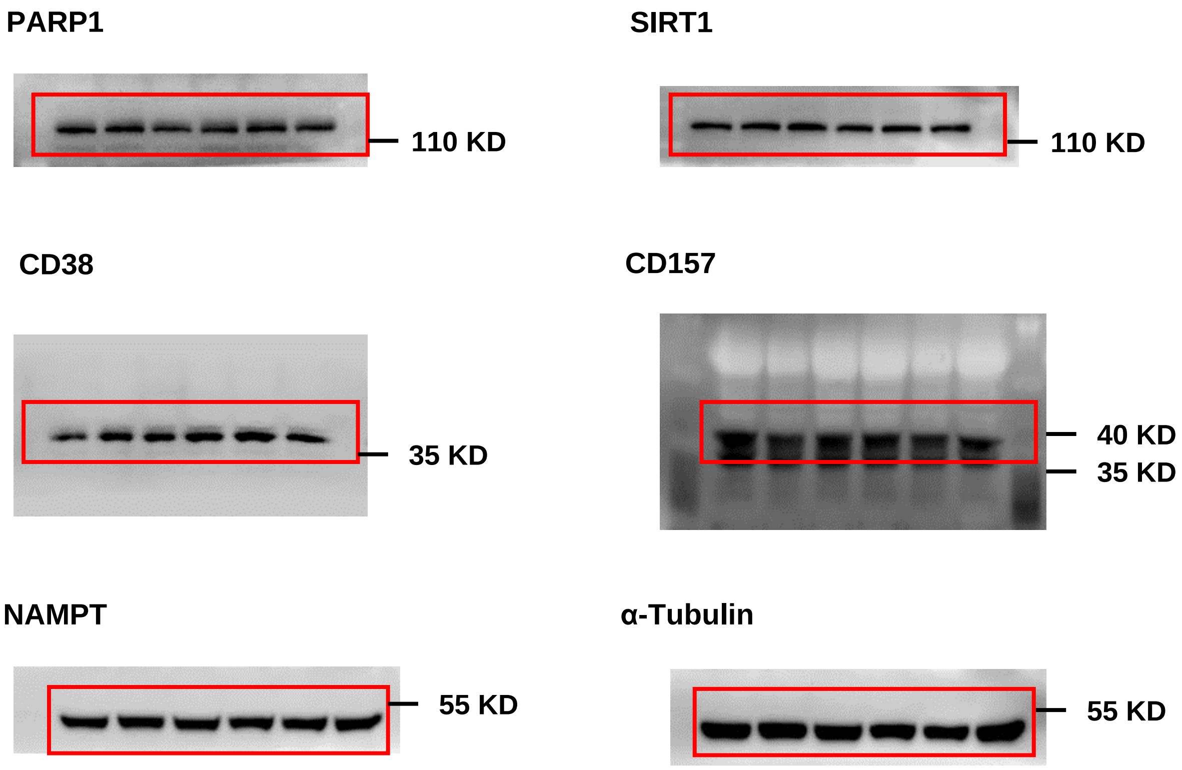
PARP1
110 KD
SIRT1
110 KD
CD38
35 KD
CD157
40 KD
35 KD
NAMPT
55 KD
α-Tubulin
55 KD

Figure 5i

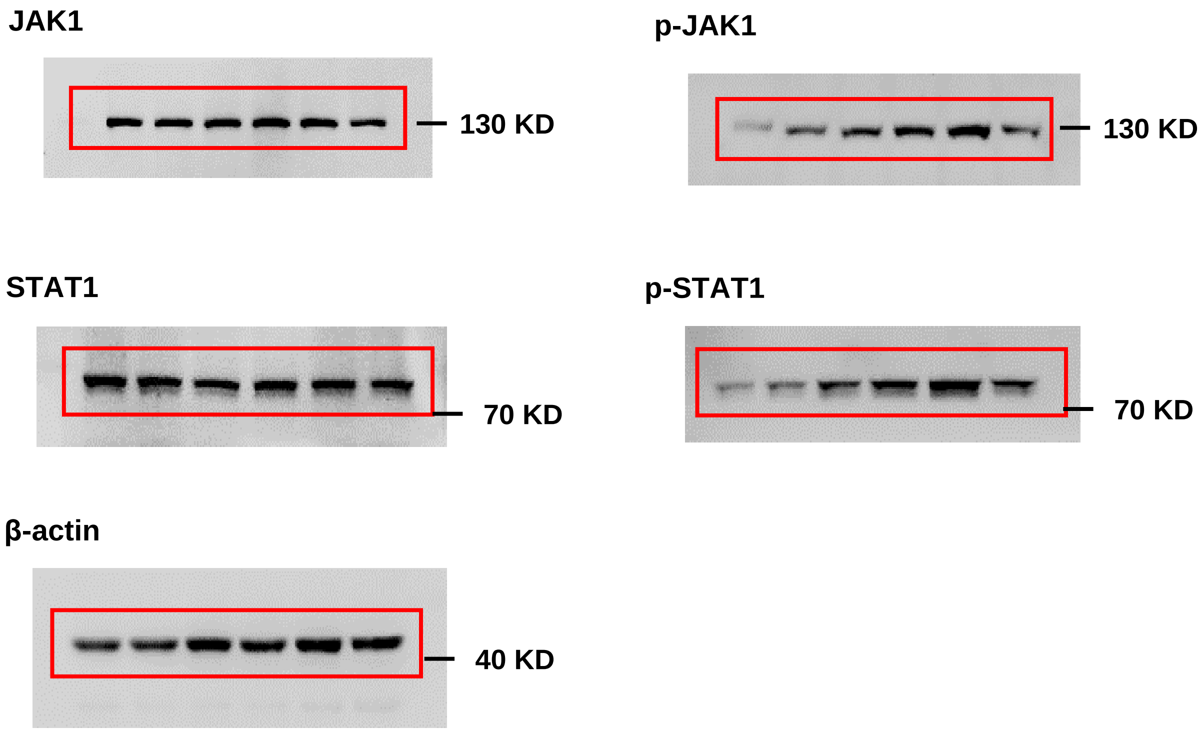

Figure5j

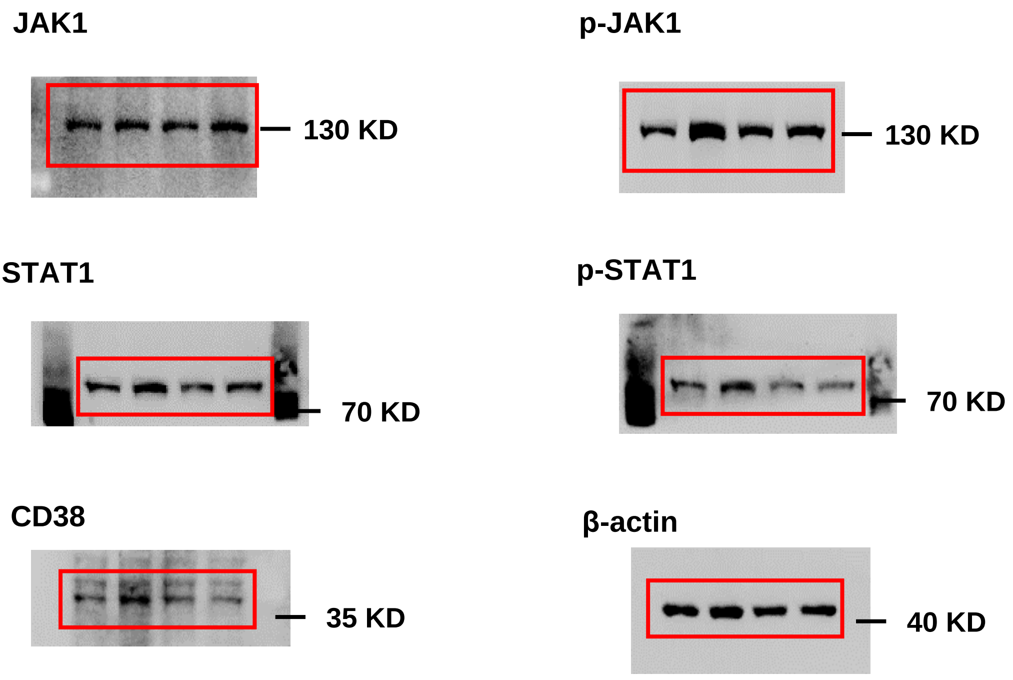
JAK1
130 KD
p-JAK1
130 KD
STAT1
70 KD
p-STAT1
70 KD
CD38
35 KD
β-actin
40 KD

Figure S4a

p-eNOS (S1177)

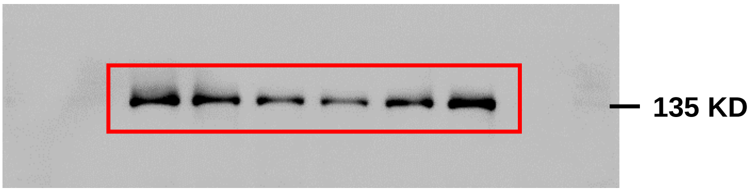

eNOS

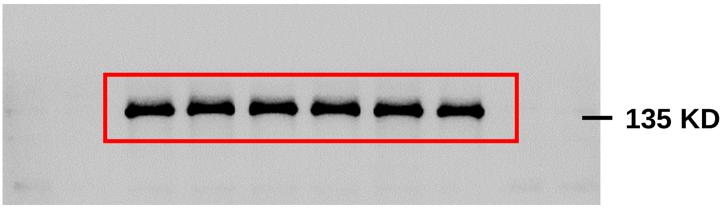

β-actin

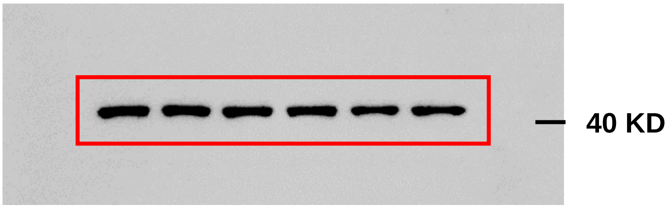

Figure S9c

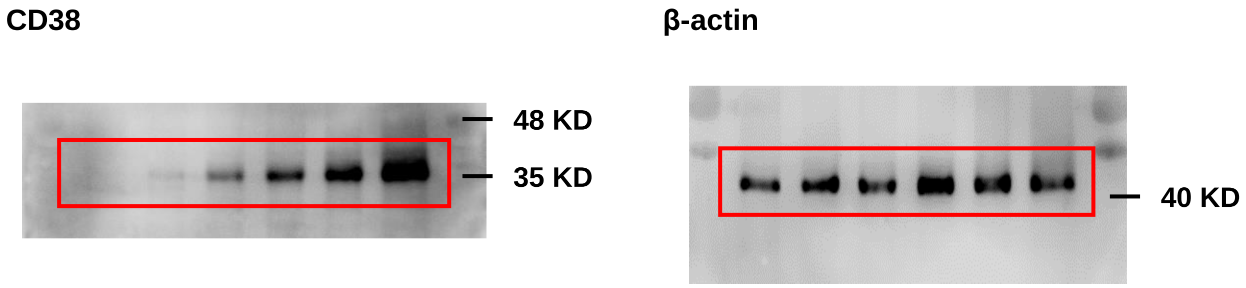

Figure S11b

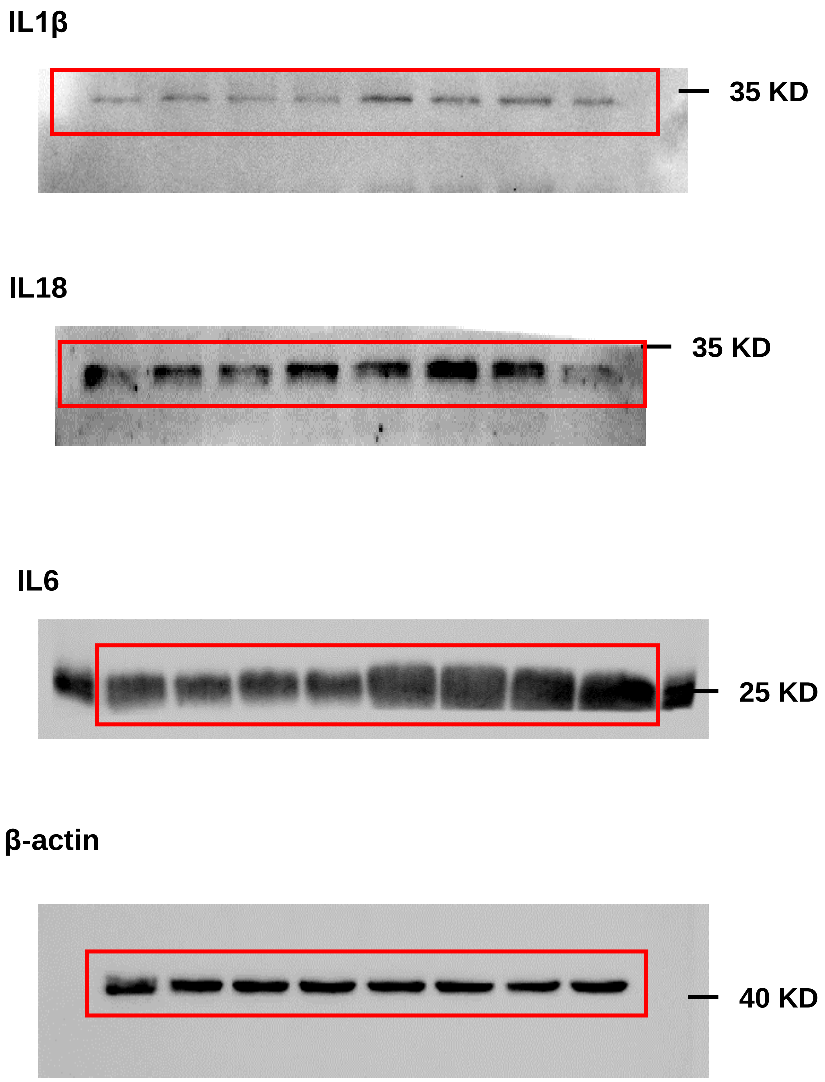

Figure S15b

CD38

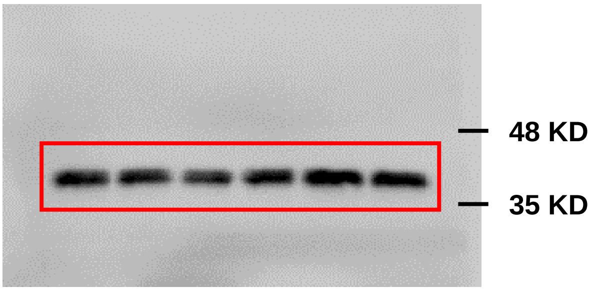

α-Tubulin

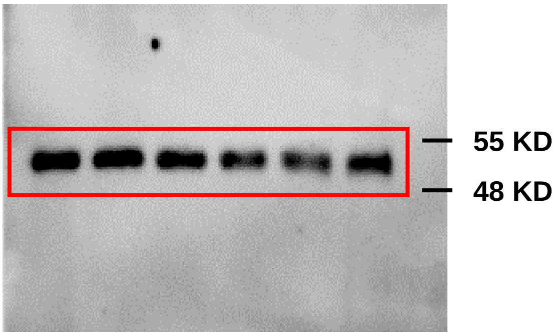

Figure S17b

IL1β

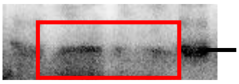

α-Tubulin

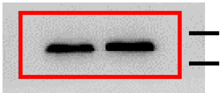

Figure S17c

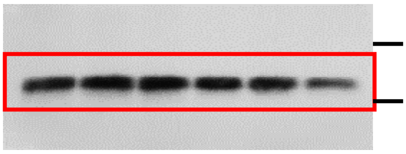

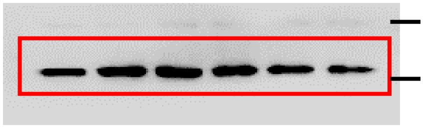

Supplement: Supplementary file 2 — original western blots [file 41392_2023_1577_MOESM2_ESM.pdf]
