# Supplementary figures and images for: NAD+ exhaustion by CD38 upregulation contributes to blood pressure elevation and vascular damage in hypertension
Source: Signal Transduct Target Ther. 2023 Sep 18;8:353. doi: 10.1038/s41392-023-01577-3 (PMC10505611; doi:10.1038/s41392-023-01577-3)

CTL

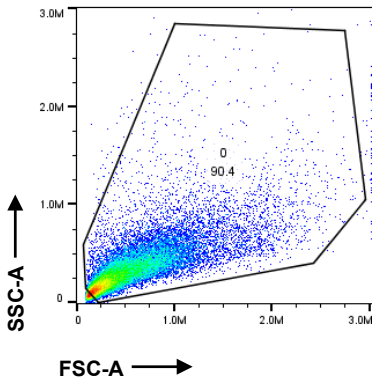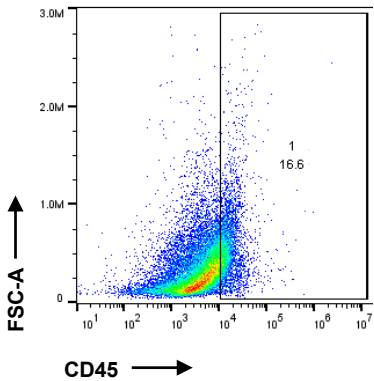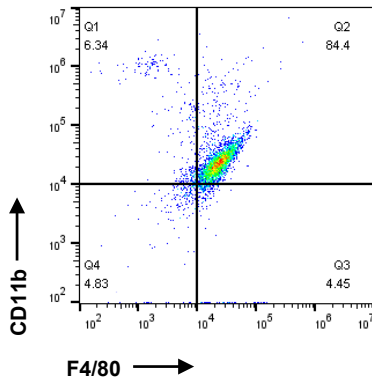

HTN

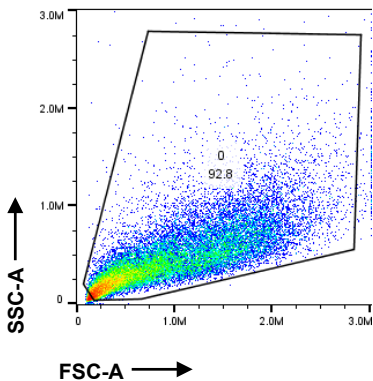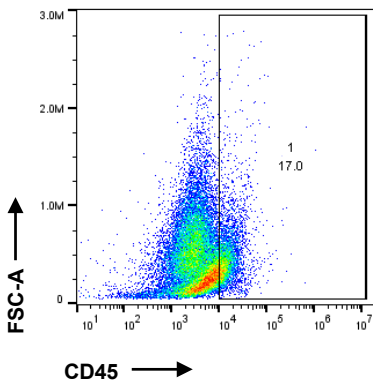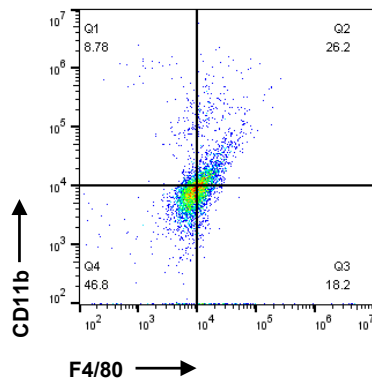

Supplement: Supplementary file 3 — SUPPLEMENTAL MATERIAL-Flow cytometry gate strategy [file 41392_2023_1577_MOESM3_ESM.pdf]
